# Matrix Metalloproteinases Shape the Tumor Microenvironment in Cancer Progression

**DOI:** 10.3390/ijms23010146

**Published:** 2021-12-23

**Authors:** Stephan Niland, Andrea Ximena Riscanevo, Johannes Andreas Eble

**Affiliations:** Institute of Physiological Chemistry and Pathobiochemistry, University of Münster, 48149 Munster, Germany; riscanev@uni-muenster.de (A.X.R.); Johannes.eble@uni-muenster.de (J.A.E.)

**Keywords:** extracellular matrix, integrins, invadosomes, matrix-metalloproteinases, metastatic cascade, MT1-MMP, therapeutic targets, tumor microenvironment

## Abstract

Cancer progression with uncontrolled tumor growth, local invasion, and metastasis depends largely on the proteolytic activity of numerous matrix metalloproteinases (MMPs), which affect tissue integrity, immune cell recruitment, and tissue turnover by degrading extracellular matrix (ECM) components and by releasing matrikines, cell surface-bound cytokines, growth factors, or their receptors. Among the MMPs, MMP-14 is the driving force behind extracellular matrix and tissue destruction during cancer invasion and metastasis. MMP-14 also influences both intercellular as well as cell–matrix communication by regulating the activity of many plasma membrane-anchored and extracellular proteins. Cancer cells and other cells of the tumor stroma, embedded in a common extracellular matrix, interact with their matrix by means of various adhesive structures, of which particularly invadopodia are capable to remodel the matrix through spatially and temporally finely tuned proteolysis. As a deeper understanding of the underlying functional mechanisms is beneficial for the development of new prognostic and predictive markers and for targeted therapies, this review examined the current knowledge of the interplay of the various MMPs in the cancer context on the protein, subcellular, and cellular level with a focus on MMP14.

## 1. Introduction

Solid tumors are complex structures of cancerous cells that are surrounded by a vascularized dynamic tumor stroma containing various non-malignant cells such as fibroblasts and myeloid cells. The prevailing conditions are similar to the inflammatory reaction during wound healing and favor angiogenesis, extracellular matrix (ECM) remodeling, and tumor cell motility [1,2]. Carcinogenesis and cancer progression depend on 10 classic hallmarks, plus four additional hallmarks for metastasis, i.e., invasive motility, modulation of the microenvironment, plasticity, and colonization [3,4,5]. Since proliferation and motility are fundamental properties of all cells, carcinogenesis is due to changes in the mutual interactions between cells and, in particular, with their ECM [6].

The events that lead to metastasis are generally similar for all types of solid tumors and rely heavily on matrix-metalloproteinases (MMPs), even if the causes for tumorigenesis diverge in different types of cancer. After a precancerous cell has undergone epithelial-to-mesenchymal transition (EMT) and has become cancerous, it breaches the basement membrane (BM) and invades the stromal ECM, which becomes possible by reorganization of integrin-containing cell-matrix adhesome structures and recruitment of ECM-degrading MMPs to them. Invasion is also promoted by tumor-induced immune tolerance and an acidic microenvironment as a consequence of an altered metabolism with lactate secretion and an upregulated proton efflux pump NHE1 in tumors [7]. Vascular dissemination and anoikis resistance are a consequence of upregulated tumor angiogenesis, attenuated apoptotic pathways, and upregulated integrin expression and phosphorylation of the Src-kinase-associated CUB domain-containing protein 1 (CDCP1) [8]. The last step in metastasis is extravasation and implantation into the pre-metastatic niche, supported by recruitment of mesenchymal stem cells and expression of chemokines and growth factors, as well as upregulated stem cell renewal pathways [9].

The tumor microenvironment (TME) is formed by cells as well as by biochemical and biophysical components of the ECM and their intricate interactions in and around a solid tumor mass. Tumor progression and metastasis depend on highly regulated and complex remodeling of the TME by pericellular proteolysis, i.e., cleavage, processing, or shedding of cell adhesion molecules, growth factors, cytokines, and kinases. This pericellular proteolysis can have both tumor-promoting and tumor-suppressive effects, which is why the mutual influence of cancer cells and the TME has become the focus of interest [10]. The ECM and its remodeling play an essential role in tumor dissemination, metastasis, and the formation of suitable metastatic niches [11,12].

Integrins as the essential matrix receptors mediate cell adhesion and enable migration through the meshwork of the ECM. Via integrin-mediated adhesion, cells perceive the surrounding ECM, react to its different properties, and interact with it with amazing specificity, which is particularly significant for cancer onset, progression, and metastatic dissemination [13].

Depending on the strength of the ECM, the migration of cancer cells is enabled or supported by the selective and specific proteolysis of certain matrix components. For example, the dense network of the BM must be broken early in the metastatic cascade. MMPs are of great importance in this context. Thus, they promote tumor development by regulating/contributing to increased invasiveness and the growth of metastatic tumors. MMP-14, in particular, as the only membrane-bound collagenase, plays a decisive role in this [14]. However, the diverse MMP functions in the TME go far beyond mere remodeling [15,16,17,18].

Various proteases, such as soluble and membrane-bound MMPs, soluble ADAMTs (a disintegrin and metalloproteinase with thrombospondin motifs) and transmembrane ADAMs (a disintegrin and metalloproteinase), cathepsins, bone morphogenetic protein 1, and Tolloid-like proteinases, as well as hyaluronidase and heparanase are involved in the formation and shaping of the TME in a variety of ways [19]. In the metastatic cascade, they are significantly involved in cancer cell invasion and metastasis. Among the various extracellular proteases, MMPs are of outstanding importance, as they considerably affect the integrity of the ECM, the phenotype, and behavior of matrix-embedded cells and tissue turnover by degrading ECM proteins and selectively releasing cell surface-bound cytokines, growth factors, or their receptors [15]. Depending on their type, these can have gelatinolytic and even collagenolytic activity towards the ECM. Of particular importance is MMP-14 (MT1-MMP), which is usually found at a low level in normal cells but can reach higher levels in cancer cells. It is the only membrane-bound collagenase and can also activate other MMPs, such as the soluble gelatinases MMP-2 and MMP-9 as well as the collagenolytic MMP-13 [20,21,22].

The TME contains numerous MMP substrates, including native fibrillar collagens and denatured gelatin as well as laminins. Stromal fibroblasts from MMP-14-deficient tumors do not degrade type I collagen, suggesting that cancer cell dissemination depends on TME remodeling by stromal cells [23]. In addition to breaking down matrix barriers, MMPs can release antimetastatic cleavage products known as matrikines from ECM components of the primary tumor.

MMPs and especially MMP-14 decisively influence the balance between cell adhesion and pericellular proteolysis of the ECM. For this purpose, the matrix receptors and proteases involved are interrelated in specialized adhesome structures called invadosomes [24]. These are found in the form of invadopodia on cancer cells and in the form of podosomes on other cells, such as endothelial cells (ECs) during angiogenic sprouting. The pericellular proteolysis that these invadosomes cause and cell adhesion are two interdependent factors that determine the TME and, thus, ultimately the prognosis for cancer patients.

## 2. Cancer Progression Is Driven by MMPs in the TME

Tumor progression and invasion of cancer cells are facilitated by numerous ECM remodeling and degrading enzymes. Among them are serine proteases, such as plasmin, plasmin activator, seprase, hepsin, and kallikreins; cysteine proteases, such as cathepsins B and K; aspartyl proteases, such as cathepsins D and E; metal ion-dependent proteases, such as MMPs and ADAMs; and others, such as heparanase, endoglycosidase, and hyaluronidase [5]. Some of them can regulate each other’s activity, and activation cascades can include endogenous inhibitors, as in the case of MMP-2, which requires tissue inhibitor of metalloproteinase (TIMP)-2 to be activated by MMP-14. Moreover, activation cascades can also be intertwined, e.g., in cathepsin(s) that activate via uPA plasmin, which ultimately leads to MMP activation [5].

More than other ECM-degrading enzymes, MMPs are pivotal in the TME [12,17]. MMPs are involved in matrix disruption, neovascularization, and subsequent metastasis and are carefully controlled in a number of ways, e.g., by TIMPs [25]. The function of the MMPs is not limited to the degradation of ECM molecules because some MMP substrates and cleavage products regulate cell growth, differentiation, and apoptosis, as well as chemotaxis, migration, and angiogenesis. Accordingly, the expression and activity of many MMPs correlate with tumor progression [5].

### 2.1. Epithelial-to-Mesenchymal Transition Depends on the Activity of MMPs

By losing apical-basolateral cell polarity and intercellular adhesion and by acquiring a migratory and invasive phenotype typical of mesenchymal stem cells, carcinoma cells undergo EMT [26]. Physiologically, it takes place during embryogenesis, wound healing, and fibrotic processes. Pathologically, it initiates cancer progression from a carcinoma in situ to an invasive tumor and metastasis [26,27]. The concomitant morphological and phenotypic changes are associated with down-regulation of E-cadherin and an up-regulation of N-cadherin, fibronectin, and vimentin [27]. The loss of tight junctions and adhesive connections in combination with an increased ECM-degrading MMP activity increases the cells’ ability to migrate and infiltrate, which is a fundamental requirement for metastasis [17,28,29]. EMT appears to vary widely between tissue and cancer types [27]. The transitions range from partial to full EMT, with carcinoma cells of intermediate EMT status being more prone to invasion and metastasis [30,31,32]. While MMP-19 is a pure mesenchymal marker like the proteoglycan asporin, other MMPs are also significantly involved in EMT [31,33,34]. MMPs with a demonstrated role in the EMT are MMPs -1, -2, -3, -7, -9, -14, and -28 [17,34,35,36,37,38,39,40,41] (Table 1). MMP-14 induces a mesenchymal phenotype in cancer and development, by cleaving BM components as well as E-cadherin [42,43,44,45,46]. MMP-14 and MMP-2 dynamically cooperate in regulating pericellular collagen homeostasis and cellular signaling processes [47,48].

### 2.2. The ECM-Degrading Activity of MMPs Is Involved in All Steps of the Metastasis Cascade

**Figure 1:**
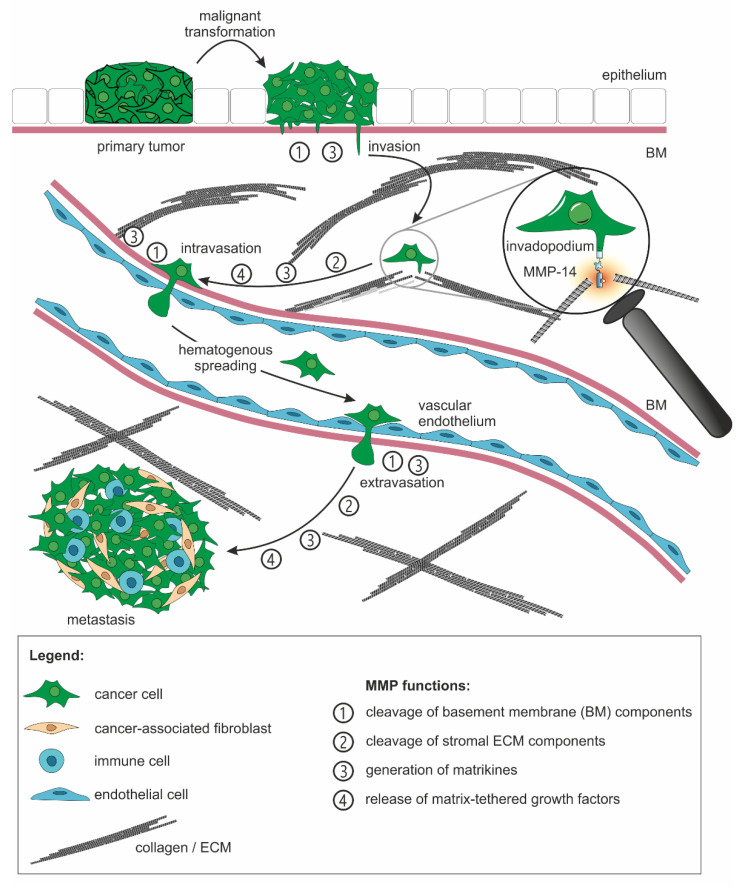
Proteolysis by matrix-metalloproteinases is crucial in every step of the metastatic cascade. A malignant tumor arises from a benign one by the acquisition of a basement membrane (BM)-breaching phenotype. To facilitate cancer cell dissemination from a primary tumor, MMPs on cancer cells cleave cell–cell adhesion molecules and are responsible for breaching the BM and invasion of the subjacent stromal ECM. They are also involved in intra-and extravasation by helping to break through the endothelial BM and the endothelium of blood vessels. Additionally, they activate ECM-tethered growth factors and release matrikines from ECM components. As the only membrane-bound collagenase, MMP-14 in invadopodia of cancer cells is of outstanding importance in the entire metastatic cascade.

In the next step of the metastatic cascade, cancer cells disseminate from the primary tumor to other tissues and organs. This is the most common cause of tumor morbidity and mortality. The invasion–metastasis cascade of hematogenous metastasis is a succession of local invasion, intravasation, survival in the circulation, arrest at a distant organ site, extravasation, initial survival in a foreign microenvironment and micrometastasis formation, and, ultimately, support by tumor angiogenesis, the formation of a secondary tumor [49]. This process depends on cell motility and invasion, modulation of the microenvironment, plasticity, and colonization [5]. In each of these steps, MMPs play a crucial role [17] (Figure 1).

Particularly, migration and invasion of cancer cells depend on the soluble MMPs -1, -3, -7, -9, -10, -11, -13, -26, and -28, and the membrane-type MMPs -14 and -16 [36,50,51] (Table 1). Above all, MMP-14 is critical to the vascular metastatic route. Remarkably, low MMP-14 levels promote invasion and vascularization in vivo, while excessive ECM breakdown by MMP-14 counteracts cell migration and tumorigenesis [52].

### 2.3. ECM Remodeling by MMPs Is Important for Tumor Angiogenesis

The vasculature provides the necessary blood supply to the tumor and facilitates the hematogenous dissemination of cancer cells. Over and above the co-option of preexisting blood vessels, tumors use all conceivable types of connection to the body’s blood circulation, with tumor angiogenesis being of particular importance [53]. Several MMPs can be induced by angiogenic factors, such as VEGF, bFGF, TGF-α and -β, and angiogenin. The latter promote angiogenesis, vasculogenesis, lymphangiogenesis, and, in particular, tumor angiogenesis [17]. In the TME, the angiogenic balance is precisely regulated by various MMPs. In particular, MMPs -1, -2, -3, -8, -9, -10, -11, -13, and -14 not only upregulate tumor angiogenesis but also can downregulate it depending on the prevailing conditions by releasing miscellaneous antiangiogenic ECM fragments or matrikines from the ECM during its remodeling [11,12,17,50,51,54,55,56] (Table 1).

Important for (tumor) angiogenesis, but not for vasculogenesis, MMP-14 acts as an endogenous suppressor in lymphangiogenesis by shedding LYVE-1 and inhibiting NF-κB-mediated VEGF-C production by macrophages [47,63,64]. During angiogenesis, tip cells at the leading edge of the neovasculature transiently regulate matrix remodeling via MMP-14, while, in immature tumor vessels, MMP-14 expression is more diffuse [55]. Moreover, MMP-14 may also shed VEGFR1 and semaphorin 4D, thereby reinforcing the proangiogenic signaling via VEGFR2 on ECs and stimulating CD8+ T-cell functions, respectively [65,66,67,68].

## 3. Molecular Biology of MMPs

### 3.1. MMPs Show Many Structural and Functional Similarities and Yet Great Diversity

**Figure 2:**
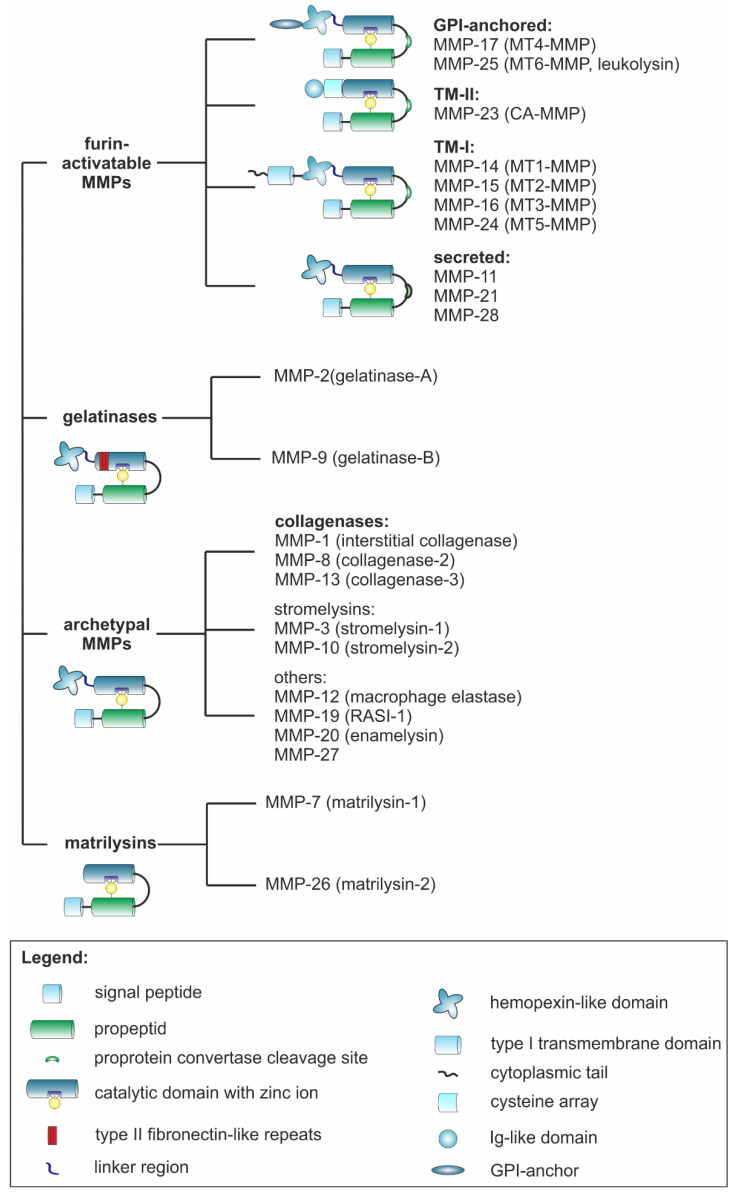
Structural and functional diversity of matrix-metalloproteinases. The 23 human MMPs are assigned to different groups according to their domain structure and substrate specificity. Most of them, except for MMPs -20, -23, and -27, are involved in processes that shape the TME [12,74,75,76].

MMPs are zinc- and calcium-dependent endopeptidases that can cleave all BM and ECM molecules. They belong, together with astacins, reprolysins, meprins, ADAMs, and ADAMTSs, to the metzincin superfamily [69]. Of 28 MMPs occurring in vertebrates, 24 are found in humans, as MMP-23 comes in two isoforms encoded by different loci (MMP-23A and MMP-23B) [70,71,72]. According to their sequence similarity, domain organization and substrate specificity, they can be classified as (1) collagenases, (2) gelatinases, (3) stromelysins, (4) matrilysins, (5) transmembrane type I, (6) transmembrane type II, (7) glycosylphosphatidylinositol-anchored (GPI-anchored), and (8) other MMPs [69,73] (Figure 2).

Despite structural and functional differences, MMPs share a similar composition of individual domains [16,71]. The generic structure of archetypal MMPs consists of an N-terminal signal peptide and a propeptide domain connected to a catalytic domain that is linked via a hinge region to a C-terminal hemopexin-like domain [77]. However, matrilysins lack a hinge and a hemopexin-like domain, while gelatinases have a characteristic insertion of three fibronectin type II repeats within the catalytic domain [77]. Furin-activatable MMPs typically contain a proprotein convertase RKRR recognition motif between their propeptide and catalytic domain [71]. In contrast to soluble MMPs, membrane-anchored MMPs are bound to the plasma membrane via a transmembrane domain or via a glycosylphosphatidylinositol (GPI) anchor, whereas MMP 23 is a type II transmembrane protein [71]. The transmembrane domain of membrane-bound MMPs is generally located near the C-terminus [71]. MMP-23 is an exception as it is kept in its latent proMMP form not by a propeptide but by an N-terminal type II transmembrane domain that contains the sequence ALCLLPA instead of the consensus motif PRCGXPD [71].

In the active center of all MMPs, three histidine residues in the HEXXHXXGXXH motif chelate a catalytically active zinc ion [71,78]. Another zinc ion and two to three calcium ions stabilize the tertiary structure of the catalytic domain and largely determine the substrate specificity [71]. Activation of proMMPs occurs via limited proteolysis by trypsin, other MMPs, plasmin, or furin-like convertases [71]. Thus, a cysteine residue within the propeptide sequence PRCGXPD, or PRCGVTD for MMP-28, is removed and makes the catalytic center of the MMP accessible to the substrate [78]. Alternatively, by oxidizing the thiol group, ROS may also render the oxidized thiol group incapable of complexing the active site Zn^2+^, thereby activating MMPs [79]. Some MMPs are also activated allosterically by a substrate molecule binding to a so-called MMP exosite outside the catalytic domain. The membrane-bound MMPs -14 and -16 are activated by intracellular cleavage by furin [80,81,82]. In addition, activation within the Golgi apparatus by the secreted proprotein convertase PCSK6 (PACE4) may occur [83]. The other membrane-bound MMPs -15, -17, -24, and -25 also contain a furin cleavage site and are believed to be activated in a similar manner prior to their membrane association [84]. Kallikrein-related peptidases can also proteolytically activate proMMPs [85].

### 3.2. MMPs Have Diverse Molecular Functions

MMPs can cleave insoluble ECM components into soluble fragments. Proteolytically, they can activate or inactivate soluble proteins and can shed and release soluble ectodomains of membrane-bound proteins as autocrine or paracrine signals [12,15]. MMP-14 shows the broadest substrate specificity of all membrane-bound MMPs, especially towards components of the pericellular ECM [47]. While MMP-14 can cleave the interstitial collagen types I, II, and III [86]. Although MMP-14 cannot directly break down type IV collagen, it can activate MMP-2 to break it down, especially during the growth of carcinomas [47,48,87]. The other membrane-bound MMPs -15, -16, and -17 can also activate pericellular proMMP-2 [88,89,90,91]. MMP-15 also cleaves type I collagen, albeit with a 100-fold lower specific activity than MMP-14, whereas MMP-16 cannot cleave type I collagen but type III [92,93]. Invasion of fibrin matrices is promoted by the fibrinolytic activity of MMPs -14, -15, and -16 [94,95,96]. Shedding of N-cadherin by MMP-17 weakens intercellular contacts and supports EMT [97].

In addition, the pericellular environment contains numerous other molecules that can be processed by MMPs (Table 1). Among these are protease inhibitors, such as α1-anti-chymotrypsin, α1-proteinase inhibitor, α2-macroglobulin, PAI (plasminogen activator inhibitor)-1, plasmin C1-inhibitor, and serine proteinase inhibitor-E2 (SERPINE2) [15,57]. Additionally, many cytokines, such as pro-IL-1β, pro-IL-8, CXCL5, CXCL9, CXCL10, CXCL11 precursor, and CXCL11, CXCL12 (SDF), pro-TNF-α, and growth factors like pro-TGF-β as well as numerous other non-matrix molecules are susceptible to cleavage by MMPs.

Even several intracellular proteins, such as cytoskeletal proteins, nuclear lamins, chaperones, regulators of transcription, translation, and apoptosis, which are involved in, e.g., transcription, translation, and carbohydrate metabolism, are reportedly MMP substrates [15,57,98]. The intracellular MMP-14 substrates that have been confirmed or identified with high confidence by degradomics include, in particular, cytoskeletal proteins, such as α-actinins -1 and -4, actin regulatory protein CAP-G (capping actin protein, gelsolin-like), actin-related protein (Arp)-2, cofilin-1, ezrin, filamins -A, -B and -C, gelsolin, moesin, plectin-1, profiling-1, tubulin-α/β, and vimentin, along with glycolytic enzymes [98]. While MMP-14 degrades the ECM scaffold extracellularly, this proteolytic cleavage of focal adhesion kinase (FAK) and signaling molecules by MMP-14 additionally promotes the disengaging of focal adhesions and thereby further enhances cell motility and thus the invasion of cancer cells [98,99].

Independently of its proteolytic activity, MMP-14 can also promote cell migration with its cytoplasmic domain [47,100]. After phosphorylation of its cytoplasmic domain, MMP-14 regulates Rac1 signaling in developing osteoclasts via p130Cas [101,102]. It also interacts with the late endosomal/lysosomal adaptor, MAPK, and mTOR activator 1 (LAMTOR1, p27RF-Rho) and, via the activation of RhoA, it promotes RhoA-dependent actin polymerization and thus cell invasion [103].

### 3.3. Matrix Metalloproteinases Are Tightly Regulated

The various MMPs must be stringently regulated to fulfill their functions in different processes without causing accidental proteolysis. While MMP expression is usually carefully regulated from the transcriptional to the post-translational level, in order to narrowly limit its spatio-temporal distribution and activity, this regulation is lost in many cancers [18,104]. Although MMP-mediated ECM remodeling and degradation play a central role in metastasis, their genes are usually not amplified, but rather dysregulated [40,105].

The proteolytic activity of MMPs is regulated on four levels: (1) regulation of gene expression by epigenetic and transcriptional control mechanisms as well as by mRNA stability, (2) compartmentalization in vesicles and membrane micro-domains in the case of membrane-bound MMPs, (3) activation from an inactive zymogen-form, and (4) inhibition of proteolysis [79].

MMP gene expression is carefully regulated via, for example, NF-κB, MAPK, and JAK/STAT signaling by cell–matrix and cell–cell interactions as well as by growth factors, glucocorticoids, cytokines, retinoic acid, interleukins, and eicosanoids [106,107,108,109]. Some MMP promoters are co-regulated due to common regulatory motifs and structural properties [106]. In addition to classifying the MMPs according to their substrate specificity, it is, hence, possible to classify them into three groups according to the regulation of their gene expression [106]. The largest group is formed by the MMPs -1, -3, -7, -9, -12, -13, -19, and -26, the promoters of which all have a TATA box (Goldberg Hogness Box) and an AP1 site, while the promoters of the second group, consisting of MMPs -8, -11, and -21, have a TATA box but no AP1 site. The MMPs- 2, -14, and -28, forming the third group, lack both, which is why they are rather constitutively expressed but are excessively expressed in some diseases [107]. The EGF receptor (EGFR) that is constitutively activated in many cancers can simultaneously activate many MMP genes as well as other genes [107]. In mesenchymal and monocytic cells, inflammatory signals like the cytokines IL-1β, TNF-α, oncostatin M, RANKL, and microbial lipopolysaccharide (LPS) are the strongest transcription activators of MMPs -1, -3, -9, -13, and -14 [107]. The expression of MMPs -2, -14, and -28 is less responsive to cytokines and growth factors than that of other MMPs [73]. Of all 23 human MMP genes, only those of MMPs -9, -14, and -15 respond to the transcription factor E2F, although all have a corresponding binding site [110].

Post-transcriptionally, trans-acting RNA-binding proteins and microRNAs regulate the synthesis of MMPs via their mRNA stability [73]. For example, MMP-14 can be downregulated by miR-181a-5p, thus inhibiting cancer cell migration and angiogenesis [111]. Similarly, miR-7 downregulates MMP-14 [112]. Furthermore, long non-coding LncRNAs regulate MMP expression. For example, the bladder cancer-associated transcript-1 (BLACAT1) downregulates the expression of MMPs -2, -9, and -14 by interacting with miR-142-5p [113]. MMPs are also subject to regulation by circular RNAs, such as circ_0007843, which binds to miR-518c-5p and upregulates MMP-2 by canceling the inhibitory effect of the miRNA [114].

**Figure 3:**
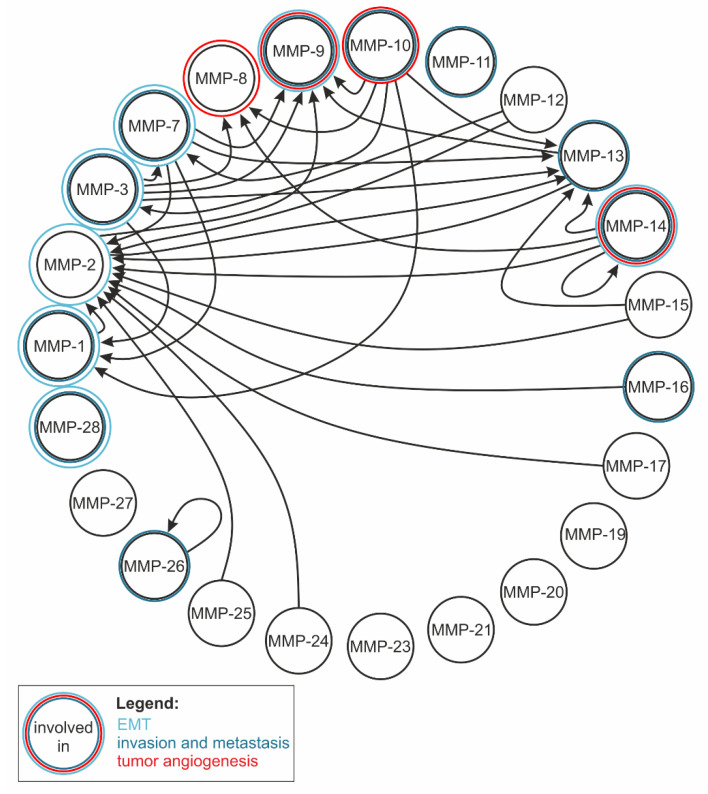
Mutual activation of the MMPs expressed in humans. In cancer, many of these MMPs are involved in EMT, invasion, and metastasis, as well as tumor angiogenesis. The mutual MMP-mediated activation of the human MPPs at the protein level is indicated by arrows. Participation in EMT, invasion, and metastasis as well as tumor angiogenesis is color coded. There is also a mutual influence on the transcription level, as explained in the text. Nearly all of the MMPs in the upper half of the figure are strongly involved in cancer progression, but, also, all the others are relevant to at least some cancers. References are in the text and in Table 1.

At the co- and post-translational levels, other regulation options are synthesis and degradation, partial proteolysis and activation by furin, phosphorylation, glycosylation, and interaction with other intracellular and extracellular proteins and lipids [12]. MMPs can mutually activate or inactivate each other in a complex network [115] (Figure 3, Table 1). For example, MMP-14 can inactivate MMP-11 in the pericellular space irreversibly by proteolytic cleavage of its catalytic domain or reversibly via TIMP-mediated inhibition [116]. There is also the possibility of proteolytic autoregulation, e.g., autocatalytic shedding terminates the MMP-14 activity on the cell surface, whereas non-autocatalytic shedding can release soluble, active MMP-14 ectodomains that are able to bind TIMP-2, thus creating a finely balanced equilibrium of soluble active and inactive enzyme fragments [117].

The proteolytic activity of MMPs, like that of ADAMs and ADAMTSs, is tightly controlled by the tissue inhibitors of metalloproteinases (TIMPs). These form 1:1 stoichiometric complexes with MMPs, with the N-terminal domain of the TIMP chelating the catalytic zinc ion in the active center of the MMP and thereby inactivating it. In contrast, TIMPs can also interact with their C-terminal domain with the hemopexin domain of MMPs and thereby activate them [118,119,120].

In particular, TIMP-1 can activate MMP-9 by forming a ternary complex of proMMP-9 with MMP-3 and TIMP-1 [121]. Likewise, TIMP-2 participates in the activation of MMP-2 by forming a complex with proMMP-2 and MMP-14 [122]. This latter mechanism also illustrates the central importance of MMP-14 as the master MMP in particular in invadopodia of invading cancer cells: Two MMP-14 molecules dimerize on the plasma membrane, with the catalytic center of one MMP-14 binding the N-terminal domain of a TIMP-2, which can interact with its C-terminus with the hemopexin domain of proMMP-2 [123]. This positions proMMP-2 so that the other MMP-14 can cleave it and release the active MMP-2 [47]. Its transmembrane domain, but not its hemopexin domain, mediates the necessary MMP-14 homodimerization [124]. In contrast, the activation of MMP-13 by MMP-14 takes place independently of TIMP-2 but depends on the hemopexin domain of MMP-13 [22,125].

MMP-14 is strictly regulated, starting with the level of transcription through post-translational modifications to its compartmentalization [126]. MMP-14 gene expression is epigenetically regulated by histone modification, chromatin remodeling, and DNA methylation-sensitive transcription factors such as SP1 [106,127,128]. The methylation status of the MMP-14 promoter, like that of the MMP-2 promoter, correlates inversely with gene expression and cell migration in vitro, with hypomethylation of the promoter and of histone H3 being associated with high levels of expression of MMP-14 and MMP-2, respectively [127].

At the transcription level, MMP-14 is also subject to strict and differential regulation with at least five transcription start sites. Unlike the promoters of other MMP genes, the MMP-14 promoter lacks a TATA box and an AP1 binding site [129]. It markedly responds to SP1 and numerous other activating transcription factors such as hypoxia-inducible factor (HIF)-2α and EGR-1, as well as E2F1, -3, and -5, and SNAI1, all of which are associated with increased malignancy in the context of various cancers [40]. SNAI1 stands out among these because it critically regulates EMT by down-regulating E-cadherin [129,130,131]. SP1 is responsible for v-Src-induced upregulation of MMP-14 [132]. The MMP-14 promoter also has a binding site for the repressing transcription factor PROX1 upstream of the main transcription start site, which significantly affects the invasiveness of cancer cells by downregulating MMP-14 [133,134,135].

In particular, ECM biomechanics and dimensionality affect MMP-14 expression [136]. Mediated by α2β1 integrin, collagen contact and mechanical forces in the TME cause an increase in the expression of MMP-14 via the transcription factor EGR-1 [137,138,139,140]. At the protein level, MMP-14 is activated by the enzymatic removal of its N-terminal propeptide by the proprotein convertase furin [82,141]. This is mediated by Golgi reassembly stacking protein 55 (GRASP55) serving as adapter while passing the trans-Golgi compartment, so that MMP-14 reaches the cell surface as an active enzyme [142].

In contrast to MMPs -1, -2, -3, and -9, whose upregulation in hypoxia is controlled by HIF-1α, MMP-14 lacks a HIF-1α binding site in its promoter [143,144,145]. Instead, MMP-14 is upregulated under the hypoxic conditions of the TME by EGR-1 and HIF-2α [140,146,147,148]. Hypoxia also induces translocation of MMP-14 to invadopodia via the small GTPase RhoA [149]. Another notably non-proteolytic feature of MMP-14, relevant in a hypoxic TME, is its ability to promote the expression of HIF target genes and the Warburg effect by activating HIFs via Munc18-1-interacting protein 3 (Mint3) and factor inhibiting HIF-1 (FIH-1) [150]. Under the hypoxic conditions of TME and depending on the increase in HIF-1α, MMP-15 as well as MMP-9 are simultaneously expressed increasingly [151,152].

MMP-14 is packaged in intracellular vesicles attached to microtubules via motor proteins. From this storage compartment, MMP-14 is delivered to developing invadopodia with the help of nesprin-2 and the dynein adaptor Lis1 [153]. Motor proteins of the kinesin superfamily (KIFs) transport the MMP-14-containing vesicles along microtubules to the cell surface. This transport is bidirectional and depends on the respective KIF. For example, KIF1B brings MMP-14 to the cell surface [126,154]. In MDA-MB 231 breast cancer cells, the transport of vesicles with MMP-14 to the cell surface is triggered by the binding of β1 integrins to collagen in a Rab8-GTPase-dependent manner [155]. Via integrin-mediated cell–matrix contacts, MMP-14-containing vesicles are directed to invadopodia at the cancer cell’s invasive front for local pericellular ECM proteolysis [14,156,157,158]. Securely stored in intracellular vesicles, activated MMP-14 can be quickly released to the cell surface when required. Contact with MMP-14-containing endosomes is mediated by the endoplasmic reticulum protein protrudin, and the Rab7-binding kinesin adapter protein FYCO1 expedites them to the plasma membrane of outgrowing invadopodia [159]. In addition, the endosomal trafficking of MMP-14 is regulated by the intracellular chloride channel 4 (CLIC4), which binds to the endosomal sorting complex (ESCRT) required for transport and also to proMMP-14, thereby promoting its proteolytic activation in lipid rafts [160]. Recycling of MMP-14 to sites of invadopodia formation is also regulated by β1 integrin-mediated Src-EGFR signaling and MMP-14 phosphorylation [161]. As fibroblasts migrate through dense collagen, MMP-14 is activated and kept at the cell surface by association with collagen-binding β1 integrins, particularly α2β1 integrin, which brings collagen to MMP-14 for proteolytic cleavage [156,157,158,162]. However, the regulation of the expression and activity of MMP-14 by collagen-binding integrins and how the topography and biomechanical properties of supramolecular collagen affect MMP-14-dependent cancer cell invasion are still not completely understood. According to the polarity of epithelial cells, MMP-14 is normally only transported to their apical surface and not to their basolateral side [163]. Stimulation with HGF, however, induces a partial translocation of MMP-14 to the basolateral side, which allows matrix remodeling and outgrowth of epithelial tubular structures into the ECM [163].

On the cell surface, MMP-14 activity is regulated by endogenous inhibitors such as TIMPs, RECK (reversion-inducing, cysteine-rich protein with Kazal motifs), testican-3, and N-Tes [164,165,166]. In particular, TIMP-3 as a guardian of the ECM regulates MMP-14, while TIMP-2 rather is involved in MMP-14-mediated activation of proMMP-2 [126,167,168]. The exposure of MMP-14 on the cell surface, but not MMP-14 gene transcription, is dependent on ADAM12, which forms with αvβ3 integrin and MMP-14 a matrix-degrading ternary complex [169]. However, the proteolytic activity of ADAM12 is not necessary for the regulation of MMP-14 activity [169].

The collagenolytic activity of MMP-14 on the cell surface is enhanced predominantly by its hemopexin domain-dependent homodimerization [124]. It is regulated by interactions with integrins, CD44, chondroitin/heparin sulfate proteoglycans, tetraspanins, pericellular MMP-14-inhibiting proteins TIMP-2, -3, and -4, and RECK [158,170,171,172]. The O-glycosylation pattern also determines the lifespan of MMP-14 and, thus, the invasiveness of cancer cells [173]. The amount of MMP-14 on the cell surface is regulated by clathrin- or caveolin-mediated endocytosis and its half-life on the cell surface is less than 30 min [174,175,176,177]. Clathrin- and caveolin-dependent internalization and recycling of MMP-14 in response to integrin-mediated matrix adhesion is controlled via FAK- and Src-mediated phosphorylation of caveolin-1 and the cytoplasmic tail of MMP-14 [14]. For clathrin-dependent endocytosis, the cytoplasmic domain of MMP-14 interacts with the coat assembly protein AP-2µ2, a component of clathrin-coated pits [176]. An important post-translational modification that regulates clathrin-dependent endocytosis of MMP-14 and, thus, MMP-14-dependent cell migration is a palmitoylation of its cytoplasmic domain close to its C-terminus [177]. Caveolin-1 antagonistically reduces the amount of MMP-14 on the cell surface by flotillin- and Rab5-dependent internalization [153,178,179]. VEGFR 2 can activate Src in ECs, and phosphorylation of caveolin-1 by Src induces the interaction of caveolin-1 with the cytoplasmic tail of MMP-14 leading to endocytosis of MMP-14 [180]. In addition to its palmitoylation, the endocytosis of MMP-14 is regulated by protein kinase C (PKC)-mediated phosphorylation of its cytoplasmic tail and promoted by the membrane-bending protein endophilin [177,181,182]. Internalized MMP-14 is subsequently degraded or recycled to the cell surface [171]. In order to get back to the cell surface after endocytosis, its C-terminal sequence DKV582 is essential [175,183]. Notably, after dissolution of podosomes, MMP-14 that remains in small membrane islets can seed the reemergence of new podosomes at these sites [184]. Another means of down-regulating the MMP-14 activity on the cell surface is its autoproteolytic or MMP-2-mediated cleavage of its catalytic domain [185,186,187].

The MMP-14 ectodomain can be shed autocatalytically and non-autocatalytically from the cell surface, either generating catalytically inactive fragments or releasing soluble, catalytically active ectodomains [187,188,189]. Nevertheless, membrane localization is a prerequisite for invasiveness because a recombinant, soluble MMP-14 does not promote cellular invasion [190].

### 3.4. MMP-14 Has a Central Role among the MMPs

As the master MMP, MMP-14 is widely expressed on many cells and is particularly overexpressed on malignant cancer cells and correlates with poor prognosis [126]. MMP-14 is essential for pericellular collagenolysis and remodeling of tumor stroma. This is a direct consequence of its involvement in the cleavage of BM and stromal ECM components, the activation of other MMPs, the release of bioactive molecules, such as TGF-β, SDF, cytokines, and matrikines, the cleavage of cell–cell and cell–matrix adhesion molecules, and other non-proteolytic functions in cancer progression. Because of its numerous functions, MMP-14 is vital for normal development. Its absence results in perinatal lethality [191]. In cells, MMP-14 knockout results in senescence, which, however, can be avoided with retinoic acid [192].

**Figure 4:**
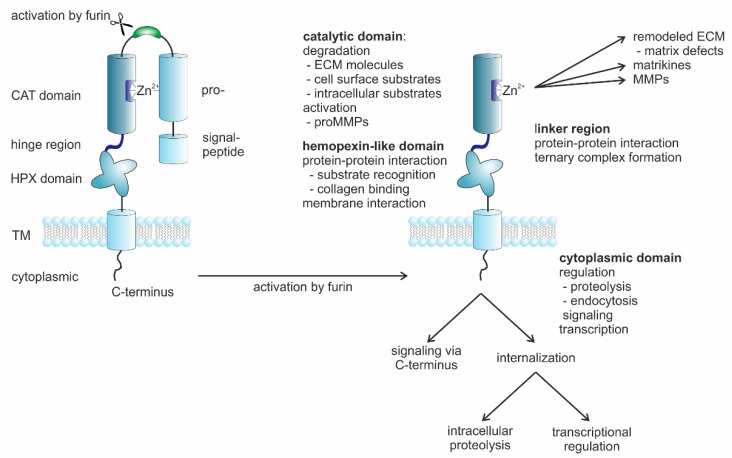
Domain structure of MMP-14 and their functions. MMP-14 is a type I transmembrane matrix-metalloproteinase that decisively determines cancer progression. MMP-14, anchored in the membrane via a C-terminal signal domain, is activated by cleavage of its cysteine-containing propeptide with a furin-like proprotein convertase, as a result of which a zinc ion is activated in its catalytic center. A hemopexin-like (HPX) domain helps in positioning the substrate for cleavage by the catalytic domain and regulates the activity by interaction with the lipid bilayer of the membrane. Its transmembrane (TM) domain and the hemopexin domain support dimerization and continue into a cytoplasmic C-terminal domain that is involved in signaling tasks.

MMP-14 (Figure 4) is a membrane-bound endopeptidase that has various pericellular activities [193]. Through the MMP-mediated cleavage of ECM components, biologically active molecules such as growth factors and cytokines including TGF-β are released or modified [194]. Further MMP-14 substrates are latent TGF-β-binding protein 1 and pro-TGF-β as well as soluble chemokines such as the stroma cell-derived factor (SDF)-1 and the monocyte chemoattractant protein (MCP)-3 [195,196]. The proteolysis of collagens and other components of the pericellular ECM in the TME activates cell signaling pathways by means of the MMP-14-generated fragments and, thus, enables cell invasion of the ECM. Moreover, MMP-14 on the cell surface can bind ligands that cause structural changes in it and affect its interactions with other cell surface molecules as well as intracellular signaling via its cytoplasmic tail.

The fibril-forming collagen types I, II, and III, in particular, and other ECM proteins such as fibronectin, vitronectin, fibrinogen and fibrin, nidogen, BM laminins, and laminin-332, which is ectopically expressed in the tumor stroma, are substrates of MMP-14 [171,197]. MMP-14 can also activate MMPs -2, -8, and -13 [61,125,141,198]. MMP-14 is the enzyme that determines the rate of collagen turnover, although other proteases including MMPs -1, -2, -8, and -13 as well as cathepsins B, K, and L can also cleave the fibrillar type I and III collagens [190,199,200].

Among the cell surface-bound MMPs, MMP-14 is the only one with collagenase activity [171]. While MMP-14 is physiologically expressed on different cell types, e.g., ECs and adipocytes [133], it is essential especially for cancer cells [122,190,200]. Here, its expression serves as an important prognostic marker, e.g., in breast cancer, as it correlates with metastatic potential [157,171,201]. In cancer progression, as in physiological developmental processes, a deficiency in MMP-14 cannot be compensated for by other MMPs. A complete knockout of MMP-14 in mice causes delayed ossification, decreased angiogenesis, severe fibrosis, and early mortality. MMP-14 knockdown by means of either RNA silencing or proteolytic shedding of the MMP-14 ectodomain diminishes cancer cell invasion [122,191,200]. In contrast, in a collagen-rich environment, the invasion of cancer cells is increased by overexpression of MMP-14 [190]. Within the TME, cancer-associated fibroblasts (CAFs) also express MMP-14 and thereby contribute to invasion and metastasis, as shown in a murine breast cancer model [23].

The HPX domain of MMP-14 plays an important role in the binding and subsequent cleavage of collagens since collagen can bind unobstructedly when blade IV associates with the lipid membrane. In addition, the interaction of the HPX domain with a lipid bilayer via its blades II and IV exposes a binding site that mediates heterodimerization with the hyaluronan receptor CD44. This, together with the observation that lipid vesicles promote the cleavage of collagen by MMP-14 ectodomains, suggests a side-by-side homodimerization of MMP-14, allowing binding to lipid bilayers, collagen, and CD44, as well as oligomerization [202].

Membrane-bound cell and matrix receptors, such as E-cadherin and CDCP1 cadherin [203,204,205], syndecans -1 and -2, and the hyaluronan receptor CD44 can also be cleaved by MMP-14 [60,206,207].

MMP-14 also increases cancer invasiveness by cleaving the N-terminal heparin-binding domain of heparin-binding EGF-like growth factor (HB-EGF) to convert it into a heparin-independent growth factor [208]. Ectodomain shedding of HB-EGF is also possible by ADAMs [209]. However, forming a complex with FGFR2 and ADAM 9 on the cell surface, MMP-14 can also cleave and inactivate ADAM9 and, thus, protect against FGFR2 shedding [210].

By shedding the hyaluronan receptor CD44 from the cell surface, MMP-14 and ADAMs, such as ADAM-10 and -17, can promote cell migration and invasion [207,211]. By shedding syndecan-1 or by proteolytically activating αv integrins, MMP-14 also increases cell migration [212,213].

Furthermore, MMP-14 is involved in the reorganization of the actin cytoskeleton through cleavage and shedding of the receptor protein tyrosine kinase PTK7, which is essential to the Wnt/planar cell polarity pathway and, hence, relevant for EMT [214].

Still a conundrum is the presence and catalytic activity of MMP-14 within the cytoplasm and nucleus [193]. Several intracellular and intranuclear substrates and interaction partners have been reported for MMP-14, such as pericentrin, a component of chromosomal centrosomes [215], centrosomal BRCA2, a DNA repair-associated tumor suppressor [216], the cytoskeletal proteins ezrin and moesin [98], and glycolytic enzymes [98,217]. MMP-14 can transcriptionally downregulate the mRNA levels of Dickkopf-related protein-3 (DKK3) in urothelial carcinoma cells, which is secreted and regulates cell invasion by interaction with Wnt signaling [218,219]. Furthermore, nuclear MMP-14 can stimulate the expression of SMAD1 via TGF-β signaling [219]. In the nucleus of a macrophage, MMP-14 may serve non-proteolytically as an epigenetic regulatory element [220] and as a transcription factor of pro-inflammatory gene expression in macrophages [220].

**Figure 5:**
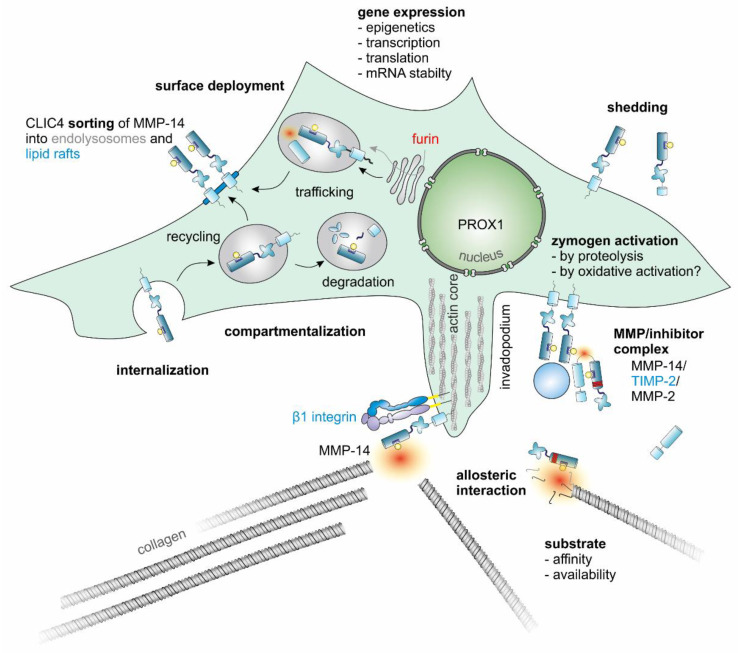
The enzymatic activity of MMP-14 is subject to complex regulation. Its gene expression is controlled by epigenetic and transcriptional factors, notably the transcription factor PROX1. In addition, MMP-14 is regulated by cotranslational removal of its signal peptide in the endoplasmic reticulum, the cleavage of its autoinhibitory prodomain in the Golgi apparatus by furin, and at the post-transcriptional level by O-glycosylation of protease-sensitive linker regions and phosphorylation and palmitoylation of its cytoplasmic domain [12].

MMP-14 is especially carefully regulated (Figure 5) because it by itself affects so many other proteins with structural, signaling, enzymatic, and non-enzymatic functions. MMP-14 is upregulated in cells by various substances such as phorbol ester or concanavalin-A and, in particular, by a three-dimensional collagenous microenvironment [47]. In contrast, the regulation of MMP-14 by tumor necrosis factor-α (TNF-α) is still unsettled [47]. MMP-14 is also upregulated by EMT-specific transcription factors such as SNAI1, TWIST, and ZEB [221]. SNAI1 also induces MMP-14- and MMP-15-dependent BM transmigration by cancer cells [222].

## 4. Cellular Adhesome Structures in the TME

**Figure 6:**
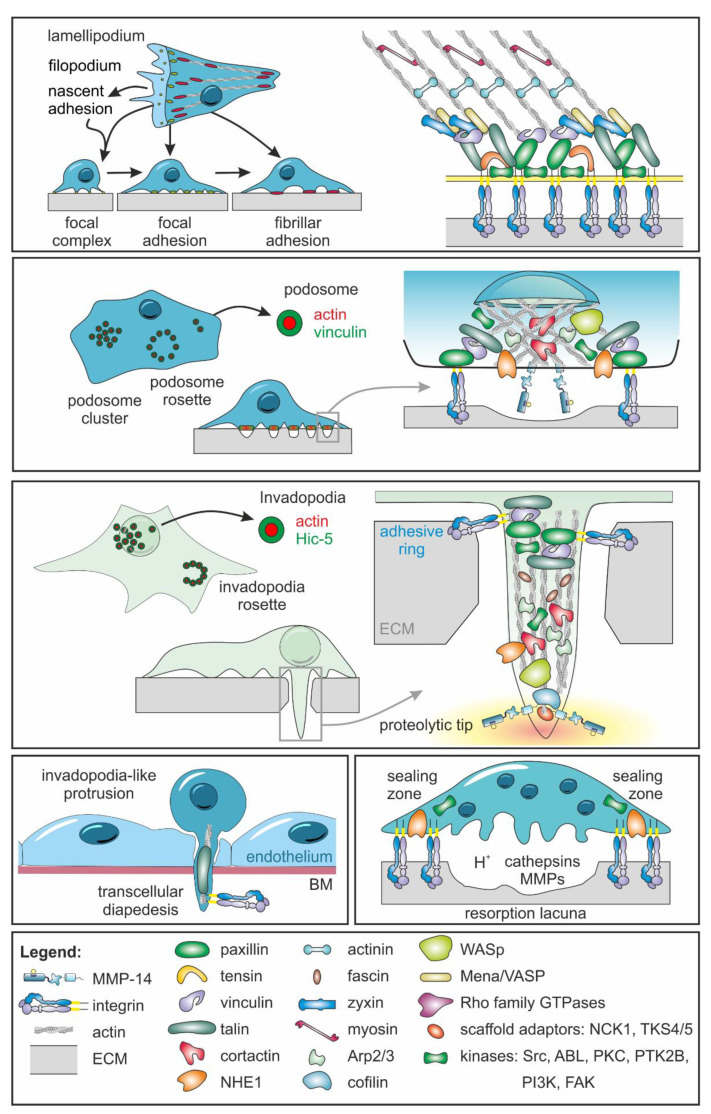
Cells employ adhesive or proteolytic adhesome structures with different composition and mesoscale organization. Details can be found in the text and in Table 2.

Integrins can perceive mechanical forces via cryptic binding sites on ECM molecules, which are made accessible by mechanical forces after being exposed through proteolytic activity, e.g., through MMPs [13]. Thus, pericellular proteolysis by MMPs, cell adhesion, and migration are interdependent processes, in which various adhesome structures are involved. The (dys)balance between adhesion to and proteolysis of the ECM by cancer and stromal cells strongly affects cancer progression [11,12,223,224]. The protein interactome of adhesomes has been meticulously elucidated [225,226,227]. Pertinent features of the most prevalent non-proteolytic and proteolytic adhesome structures (Figure 6) are summarized in Table 2.

### 4.1. Using Varied Adhesome Structures, Cells Can Interact Differently with the ECM

Dysregulation of normal cell growth in cancer does not only affect cancer cells but also stromal cells [224,261]. Cell growth and migration depend inter alia on the interaction of these cells with the ECM, mediated by adhesion receptors and other adhesome components [262]. Cell adhesion is mediated via integrins. They reinforce the connection of the ECM to the cytoskeleton [263]. However, their role depends on the type and stage of cancer [264,265]. They are composed of 18 α and 8 β subunits in mammals that form 24 different heterodimers [266]. These heterodimers bind to a variety of ligands, which are divided into Arg-Gly-Asp (RGD) binding and non-RGD binding receptors [267]. Integrins are characterized for having three different conformational changes. Inactive integrins with low affinity for the ECM ligand adopt a bent-closed conformation, whereas active integrins, capable of binding ligands with increasing affinity, adopt an extended-closed and extended-open conformation [268]. These conformations depend on activators, inactivators, and ligand interactions of the integrins [269].

Signaling via integrins is bidirectional: inside-out and outside-in signaling [270]. In inside-out signaling, intracellular integrin activators, e.g., talin and kindlin, induce integrin activation by binding to the integrin β subunit tail [270,271]. In outside-in signaling, signals originate from ECM ligand binding to the integrin extracellular domain and are conformationally conveyed to the cytoplasmic domain [272,273,274,275]. Thus, mechanical signals in the cell could be transduced via conformational changes either outside-in or inside-out [271,276]. These signals regulate cell migration, differentiation, proliferation, survival, and cytoskeletal organization [272,277].

The adhesome comprises the entire network of structural and signal proteins that are involved in the regulation of cell–matrix adhesion [225,227,278]. While several hundred different proteins occur in various adhesome structures, the core adhesome centered around the integrin consists of around 60 proteins that are common to all adhesome structures [278,279]. Among them are tyrosine and serine/threonine kinases, phosphatases, guanine nucleotide exchange factors, GTPase activating proteins, E3-ligases, and proteases that regulate adhesion through post translational modifications of many of its structural and scaffolding proteins [280]. Varying in size, shape, distribution, dynamics, and molecular components, adhesome structures show plasticity and enable cells to perceive, adapt, and respond to differences in the extracellular environment. According to morphological and molecular criteria, adhesome structures can be divided into focal complexes, focal adhesions, and fibrillar adhesions, with their corresponding correlates in three-dimensional matrices, as well as invadosomes [281]. They also have different functions. Some predominantly serve mechanical anchorage of cells, while others possess proteolytic activities. It is important to understand when and how a cell switches its adhesome type, e.g., from focal adhesions to invadosomes [282]. Moreover, albeit having ECM-cleaving activity, invadopodia protruding into the ECM can expand matrix defects and cavities by means of contractile forces without using proteases [283].

### 4.2. Focal Complexes Are Formed as the First Adhesive Matrix Contacts

Cells form different membrane protrusions to explore the surrounding ECM, such as filopodia [284] and lamellipodia [285]. MMP-14 has been reported to be also localized to filopodia [286] and lamellipodia, where it indirectly associates with the actin cytoskeleton by binding with its HPX domain to the hyaluronan receptor CD44 [287,288,289,290]. These membrane protrusions are mechanically stabilized by adhesive matrix contacts, to which several adhesome components are recruited, e.g., regulators of lamellipodial cell migration, Rac, WASp, Arp2/3, arpin, lamellipodin, WAVE, Mena, and Ena/VASP family members. They also control actin dynamics in both endocytosis processes and invadopodia [291]. Upon initial integrin-mediated contact to the ECM, more integrins can be recruited to the contact site, forming a nascent cell adhesion contact or focal complex. Such focal complexes, developing from nascent focal complexes with a diameter of 0.25 µm and a half-life around 60 s, can mature into larger and more stable focal adhesions and further into fibrillar adhesions [247,281].

### 4.3. Focal Adhesions and Fibrillary Adhesions Allow Force Exertion

Cells migrating on a two-dimensional surface form lamellipodia, in which initially formed focal complexes are converted into focal adhesions for force transmission. Therefore, lamellipodia can be considered as the engine that propels a migrating cell. In addition, proteases, such as MMP-14, MMP-2, uPA(-R), and seprase, can localize in lamellipodia with structures containing integrins or paxillin [292] that, thus, are distinct from focal complexes or adhesions [292,293,294,295]. Mechanosensors such as actin polymerization-inducing Ena/VASP-like (EVL) promote durotaxis and reinforce focal adhesions [296]. MMP-14 can be recruited to focal adhesions due to the association of its cytoplasmic domain with a FAK-p130Cas complex [297]. However, the cytoplasmic domain of MMP-14 is dispensable for the degradation of the underlying matrix and localization at focal adhesion sites [124,298,299,300]. In contrast, a so-called MT-loop of eight amino acids in the catalytic domain of MMP-14 is critical for its localization at focal adhesions when the ECM is degraded there [300].

Focal adhesions can grow into fibrillar adhesions, which are involved in ECM remodeling, especially in fibroblasts adhering to a fibronectin matrix [301]. Moreover, tumor cells tend to realign the meshwork of collagen fibers in the ECM of the TME by forming fibrillary adhesions [199,302,303].

### 4.4. Podosomes Coordinate Cell Adhesion with Focal ECM Degradation

At the ventral side of the Ras-transformed fibroblasts, vinculin and α-actinin can rearrange from focal adhesions into circular membrane protrusions, which, on the one hand, mediate cell adhesion and were, therefore, referred to as podosomes, and, on the other hand, proteolytically degrade the ECM, which is why the term invadopodia was coined soon afterwards [251]. It is controversial whether podosomes majorly differ from cancer cell invadopodia and/or whether invadopodia are merely dysregulated podosomes. Therefore, both are grouped under the generic term invadosomes [304].

Podosomes are conical, actin-rich structures on the surface of normal non-cancerous cells that serve both cell–matrix adhesion and localized ECM degradation. They are usually between 0.5 µm and 2.0 µm in diameter and length. With a lifespan of just a few minutes, they are more dynamic than invadopodia [305,306]. While invadopodia are characteristic of invading cancer cells, podosomes are involved in normal biological processes such as overcoming tissue barriers by immune cells or in bone remodeling [307]. Hence, podosomes are found on macrophages, dendritic cells, and osteoclasts, but also on ECs, vascular smooth muscle cells, and Src-transformed fibroblasts [308]. Podosomes as well as similar structures in Ras-transformed fibroblasts, ECs, macrophages, and dendritic cells contain MMP-14 [47,251]. If, despite having specific characteristics of podosomes, the adhesome structure deviates from the consensus, it is sometimes referred to as a podosome-like structure [309].

Podosomes coordinate the degradation of the ECM with cell movement and, thus, regulate migration in the microenvironment, which is crucial for processes such as embryonic development, wound healing, and inflammatory reactions [251]. Podosomes are also involved in mobilizing mesodermal progenitor cells (MPCs) in the bone marrow that differentiate into endothelial or mesenchymal cells [310]. As a multi-purpose organelle, podosomes are additionally involved in weakening tight junctions of ECs by sequestering zonula occludens’ proteins and in the fusion of cells, such as osteoclasts, myoblasts, and macrophages [311,312,313].

The turnover of the podosomal actin core, which is carefully regulated by Arp2/3, WASp, cortactin, and numerous other actin nucleators, polymerization activators, actin-binding, and cross-linking and scaffolding proteins as well as kinases and small G proteins, takes seconds [314]. Confined by a cap structure, from the dense actin core, a set of unbranched filaments protrudes radially towards a ring of adhesion plaque proteins, such as paxillin, vinculin, talin, and integrins, as well as p21-activated kinase 4 (PAK4), while others extend to neighboring podosomes [315,316,317]. In the ring complex, integrins and integrin-associated adhesion plaque proteins connect the cytoskeleton to the ECM [305,318,319,320]. The cap essentially regulates podosome contractility [233,321]. A detailed explanation of the podosome architecture and mechanics can be found in [234].

The formation of podosomes depends not only on the structure and composition of the underlying substrate and the presence and distribution of specific integrin ligands, but podosomes also act as mechanosensors [250,322]. A higher matrix stiffness extends the lifespan of podosomes and results in a closer spacing between them [323]. With their integrin-containing ring structure, podosomes generate mechanical forces in the range of several pN [324,325].

The major cellular transducer of mechanical signals in a cell is YAP, which responds to a broad range of signals, such as shear stress, cell shape, and ECM rigidity [326]. Through ECM remodeling, MMP-14 can promote integrin clustering that activates a β1-integrin/Rho GTPase signal cascade, which leads to the nuclear translocation of the transcription coactivators YAP and TAZ [327]. In addition, YAP signaling is controlled via its ubiquitination and subsequent degradation by the ubiquitin domain-containing protein 1 (UBTD1), associated with β-catenin at cell–cell adhesion sites [328].

### 4.5. Invadosomes Are Both Adhesive and Proteolytic Structures

The umbrella term invadosomes combines the adhesome structures, invadopodia and podosomes, both of which allow the focal breakdown of ECM [232]. According to an updated definition proposed by Cambi and Chavrier, a mesoscale organization for podosomes and the location underneath the nucleus for invadopodia are important criteria for classification, in addition to minimal structural requirements, such as the incorporation of cortactin and a core of F-actin, as well as the ability to degrade the ECM [153,329,330,331].

**Figure 7:**
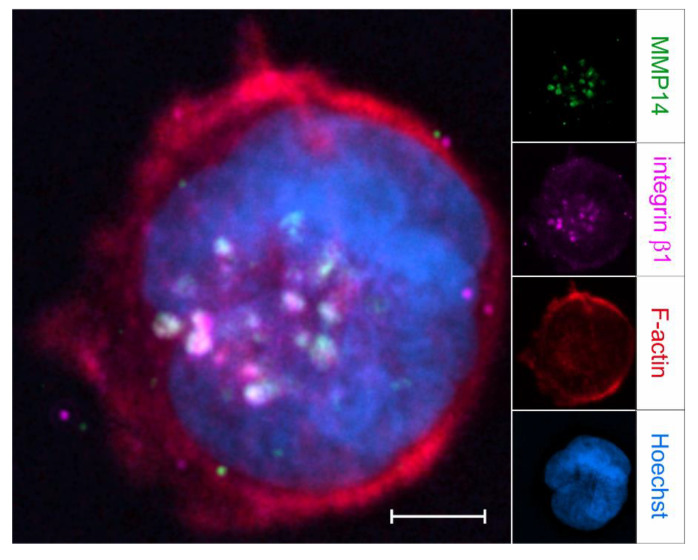
Invadopodia beneath the nucleus of an MDA-MB-231 breast cancer cell in a type I collagen matrix are both proteolytic and adhesive. MMP-14 is immunostained in green, active integrin β1 in magenta, and F-actin in red. The nucleus is counterstained in blue with Hoechst 33,342 dye. Scale bar: 5 µm.

Invadopodia (Figure 7) have an actin core surrounded by actin-binding proteins, scaffold proteins, and adhesion molecules [251,304,332]. They are formed in three successive steps, starting from initiation, through stabilization to maturation [306,332]. Invadopodia formation is largely initiated via PI3K by Src family kinases after activation by EGF, TGF-β, or PDGF, whereupon plasma membrane buds are formed [251]. In contrast to focal adhesion-mediated cell–matrix interaction, which is associated with myosin-II-mediated cell functions, an increase in Src activity shifts the cellular adhesome towards the formation of invadosomes [333]. The Src kinases then phosphorylate invadopodia proteins, such as TKS5, synaptojanin-2, and tyrosine-protein kinase ABL2 (Arg) [334]. Through their phosphorylation, N-WASp is recruited to invadopodia, which, by means of Arp2/3, promotes actin polymerization and, thus, invadopodia elongation [334]. Subsequently, the scaffold protein TKS5 interacts with its phox homology (PX) domain with the phospholipid PI(3,4)P2, whereby the invadopodium core is anchored to the plasma membrane and the newly formed invadopodium is stabilized [306]. A sustained actin polymerization, regulated by cofilin, fascin, tyrosine-protein kinase ABL2, and mDia2, together with the recruitment of MMP-14 as well as MMP-2 and MMP-9, results in mature invadopodia, in which an actin core is surrounded by a ring of adhesion proteins, such as integrins, paxillin, vinculin, a-actinin, and HIC-5 (TGFB1I1) [333,334]. Their lifetime and the spatiotemporal secretion of MMPs is controlled by Rho GTPases [333].

By the regulation of this intracellular trafficking, activated MMP-14 is kept ready in intracellular vesicles clustered around the centrosome and close to the cell nucleus for ‘digest on demand’ [153]. MMP-14 is targeted to the base of invadopodia by means of late endosomes and lysosomes [159,335]. Fusion of MMP-14-bearing vesicles is mediated via synaptotagmin VII [159]. Instead of vesicles, endosomal tubules can form in a JIP3/4- and WASH-dependent manner to deliver MMP-14 to the tip of invadopodia [336,337,338].

β1 integrin signaling is crucial for the formation of invadopodia in various cancer cell types [251,339,340]. Recycling of MMP-14 at invadopodia formation sites is controlled by the phosphorylation of MMP-14 via the β1-integrin-Src-EGFR signaling pathway [161]. β1 integrins control SNARE-dependent delivery of Src and EGFR to sites of invadopodia formation [341]. Additionally, lamellipodia formation and membrane ruffling, as well as focal adhesion kinase signaling and Src-regulated focal adhesion turnover, depend on SNARE-mediated membrane traffic [342,343]. Likewise, the exposure of MMP-14 on the surface of invadopodia depends on SNARE complexes containing SNAP23, VAMP3, syntaxin13 or SNAP23, syntaxin4, and VAMP7 [344,345,346]. Additionally, MMP-14 can be delivered into the pericellular ECM by means of exosomes, which are released by fusion of late multivesicular endosomes with the plasma membrane [347]. Active MMP-14- and β1-integrin-containing exosomes can activate proMMP-2 and promote ECM degradation [348]. Thus, SNARE regulators may be targets for the development of antimetastatic therapies for cancers with invadopodia involvement [346].

TKS5, integrin, F-actin, cortactin, and MMP-14 were identified as prognostic markers related to invadopodia and their targeting curbs breast cancer metastasis [349]. The scaffold protein TKS5 correlates with invasiveness. Its overexpression, e.g., in prostate cancer, promotes the formation of invadopodia and matrix degradation in a Src-dependent manner [350]. Additionally, in gliomas, increased TKS5 expression correlates with poor patient prognosis [351]. In a mouse model of lung adenocarcinoma, non-metastatic tumors expressing a short isoform of TKS5 could be turned into invasive ones by the expression of a long TKS5 isoform found in invasive tumors [352]. Conversely, the knockdown of TKS5 in human mammary epithelial (HMLER) cells decreases tumor invasion and metastasis [353].

Cortactin is concentrated in invadopodia of head and neck as well as breast cancer cells [354]. For instance, microinjections of anti-cortactin antibodies reduce the ECM degradation at invadopodia in MDA-MB-231 cells [355]. On the other hand, an increase in the ECM degradation and invadopodia number occurs after cortactin overexpression of HNSCCs and MDA-MB-231 [356,357]. Cortactin and TKS5 associate and correlate in their expression with different proteases, which are related to invadopodia, including MMP-2, MMP-9, and MMP-14. The exact mechanisms of how the cortactin and TKS5 regulate the MMPs’ secretion are still unknown [358,359,360].

Invadopodia are highly dynamic structures [250,361,362]. Nevertheless, they have a longer half-life than podosomes. Ras GTPase-activating-like protein (IQGAP1) can control the lifespan of invadopodia by keeping Cdc42, which plays a crucial role in the formation of invadopodia, active [14,363]. With a length of more than 2 µm and a width between 0.5 µm and 2 µm, they are also larger than podosomes [251]. Accordingly, invadopodia penetrate deeper into the ECM than podosomes and degrade the ECM to a greater extent [304]. Invadopodia persists for many hours, whereas podosomes have a life span of a few minutes [364,365]. The number of invadopodia and podosomes depends on the cell type. Nonetheless, it has been reported that there are around seven invadopodia and up to hundreds of podosomes per cell [249,366,367]. Invadopodia are preferably formed under the cell nucleus, which serves as a mechanical abutment to increase their propulsive power (Figure 7) [330]. Investigating the role of focal adhesions in invadopodia stability, so-called focal adhesion rings containing vinculin and talin were found to oscillate just like cortactin with a period of 10 min, demonstrating that invadopodia are highly dynamic structures in which ECM adhesion and proteolysis are finely tuned [12]. For this purpose, invadopodia as well as podosomes serve as local storage depots for proteases near and at the cell surface [10].

It must be noted that the general invadosome ring model needs to be studied further using three-dimensional fluorescence microscopy. A ring organization is not mandatory for the constitution of the core. In fact, invadosomes can be organized into six different clusters depending on the cell type: linear, array, single, rosette, belt, and ring [256,257]. This is the case, e.g., in trophoblast podosomes, which form an atypical core [368].

Invadopodia are the equivalent of podosomes on malignant cells. They are equipped with proteases that can break down the pericellular ECM. Although the serine protease seprase was originally identified as invadopodia-localized protease, the major protease responsible for ECM degradation by invadopodia is MMP-14 [157,251,360,369]. The localization of MMP-14 in invadopodia depends on its cytoplasmic domain [360], which is also necessary for the N-WASp-mediated localization of MMP-14 in invadopodia-like structures [370]. Other major constituents of invadopodia are, in addition to β1 and β3 integrins, an F-actin scaffold; cytoskeletal regulators, such as cortactin, fascin, Arp2/3, cofilin, WASp family members, Mena/VASP, paxillin, and Rho family GTPases; and the scaffold and adaptor proteins NCK1 and TKS4/5, as well as various kinases, such as Src, ABL kinases, PKC, FAK, PTK2B, and PI3K [371].

Invadosomes also have a dynamic life cycle [282]. Cells of breast acini probe the ECM with non-proteolytic actin microspikes, which, as functional precursors of invadopodia, widen pores in the collagen IV meshwork with force-transmitting focal adhesions and initiate BM penetration [372]. In cells disseminating into the ECM, such non-proteolytic microspikes are then converted into proteolytically active invadopodia containing MMP-14 [372]. The formation of invadopodia is stimulated by ECM rigidity, degradation of ECM molecules, and growth factor signaling [361,373,374,375,376,377]. As invadopodia mature, they elongate and expand through actin polymerization and cortactin-dependent actin branching, and lysosomal and late endosomal vesicles containing MMP-14 fuse with the invadopodial plasma membrane [14,159,378]. By recycling from endosomal pools, MMP-14 is incorporated into invadopodia, where a high concentration of MMP-14 is maintained by anchoring MMP-14 to the invadopodial actin/cortactin core [14,370]. Via the protrudin pathway, MMP-14 is brought to the base of invadopodia and, by means of the endosomal tubulation machinery, into the narrow invadopodia core and to the tip of invadopodia [159]. MMP-14 remains restricted to the invadopodia, with dystroglycan and ECM adhesion proteins forming a barrier against lateral diffusion at the invadopodia base [226,378]. During cancer cell invasion, cells switch back and forth between periods of integrin-mediated migration and invadosome-mediated matrix degradation and passage [379,380]. Several non-receptor tyrosine kinases (NRTKs) jointly regulate invadopodia formation and function and, thus, control the balance between local tumor growth on the one hand and invasiveness and spread of tumor cells on the other hand [381]. FAK counteracts the formation of invadopodia by withdrawing Src from invadopodia and recruiting it into focal adhesions [382]. In contrast, PTK2B is responsible for recruitment and activation of Src and also for the phosphorylation of cortactin [383]. While Src promotes the assembly of invadopodium precursor structures, which it stabilizes by phosphorylation of TKS5, PTK2B phosphorylates cortactin after activation by integrins or other signals, such as EGFR, both directly and indirectly via Src and the Abelson-related gene tyrosine kinase ABL2 [350,384,385,386]. This tyrosine-phosphorylated cortactin then activates several signaling pathways that result in actin polymerization, ECM degradation, and docking and secretion of exosomes, thus promoting invasion and dissemination of cancer cells [387].

It is still open whether invadosomes and podosomes share a similar mechanosensory mechanism. In the three-dimensional ECM meshwork, mechanosensing and actin-based cell protrusions are controlled by p21-activated kinase 1 (PAK1) [388]. The mechanosensitive ion channel Piezo1, which activates in ECs the MMPs -2 and -14 and promotes translocation of MMP-14 to the plasma membrane, is highly expressed in breast cancer and gastric tumor cell lines where its inhibition and knockdown impede cell motility and migration [389,390,391].

Prior to their extravasation, metastatic cancer cells and T-cells can project invadopodia-like protrusions (ILPs) at endothelial junctions into the extravascular stroma, likely to scout a potential site for diapedesis [392]. This could explain why some cancers preferentially metastasize into different organs [243]. Invadopodia-like structures, however, are not yet characterized for localized accumulation or secretion of proteases [243,393]. Although proteases appear to be dispensable for transcellular pore formation, proteases on ILPs (1) could influence the local density of adhesion molecules and chemokines and, thus, the adhesion and signal dynamics at the endothelium, and (2) might be involved in BM penetration [243]. ILPs also protrude forcefully from the bottom of T-cells, thereby indenting the endothelium and creating close contacts to ECs in so-called ILP footprints or podo-prints [235,394].

To degrade larger areas of the ECM, larger, rosette-shaped aggregates of invadopodia can arise [333]. HIC-5, a scaffold protein closely related to paxillin, is essential for the formation of such invadopodia rosettes [333].

Another type of invadosomes is the so-called linear invadopodia. They differ significantly in their structure from invadopodia in the conventional sense, but also enable matrix remodeling by ECs and embryonic and cancer cells [395]. Associated with collagen I fibrils, they have a linear organization of invadosome-typical scaffold proteins TKS5, Src, WASp, Arp2/3, and cortactin and have proteolytic activity thanks to MMPs -14 and -2. However, they lack integrins and other proteins such as vinculin and paxillin. Instead, they are regulated via DDR1 as a collagen receptor and CDC42 and its guanidine exchange factor Tuba [396].

### 4.6. A Sealing Zone Surrounds the Resorption Lacuna of Osteoclasts

Podosomes are also instrumental in bone remodeling by osteoclasts, large multinucleated bone cells that resorb bone matrix [365]. During the maturation of osteoclast precursors, groups of podosomes assemble into higher-order ring- and belt-like structures that eventually fuse to form a band-shaped sealing zone that delimits and, due to further tightening of its F-actin network, seals the resorption lacuna [397,398,399]. Bone remodeling and homeostasis are controlled by several MMPs and cathepsin K [400]. For example, bone catabolism depends on the cooperation of MMP-9 and MP-14 secreted by osteoclasts [401]. The bioavailability and activity of TGF-β and BMPs is also regulated by MMPs [402].

In contrast to the resorption zone, which is acidified by the vacuolar ATPase and the chloride-proton antiporter CLCN7, the sealing zone contains NHE-1 and, in this respect, is similar to invadopodia [236]. Recruited by Src-dependent cortactin phosphorylation, NHE1 exchanges here locally extracellular Na^+^ for intracellular H^+^. The resulting increase in intracellular pH leads, via clustering of phosphatidyl-inositol-4,5-bisphosphate, to an activation of cofilin, thus regulating the actin-dependent pushing force of the cell protrusion [403,404]. Src is required for the organization of the cytoskeleton in osteoclasts and is, therefore, indispensable for efficient bone resorption [405].

### 4.7. Invading Cancer Cells Can Cleave the ECM at Belt-Like Compressions That Impede Cell Migration

After initial cleavage of the impeding ECM meshwork, cancer cells move forward. However, along their way through tight interstitial spaces, they are severely squeezed in this process, showing a belt-like compression [292,406]. If the space between the collagen fibers in a dense ECM becomes increasingly narrow, the cell nucleus deforms to a certain extent, so that the cell can squeeze through narrow spaces down to 25 µm^2^ in cross section [406]. However, when the ECM meshwork becomes too dense for migration, the cell locally degrades the ECM structures at the constriction site and generates small trail-like matrix defects [407,408].

## 5. MMPs and TME: More Than a Hit-and-Run Relation

### 5.1. MMPs Play an Essential Role in the Remodeling of Tumor Stroma ECM

The ECM, which is much more than a mere structural framework and which regulates and fine-tunes practically all cellular processes from cell adhesion and migration to survival and proliferation, is usually modified biochemically, biomechanically, and topographically in cancer [11,19]. It is subject to remodeling by cancer cells and other cells of the tumor stroma, such as CAFs [409]. A frequent notion describing the TME is that a tumor resembles a wound that never heals [410,411]. In fact, both show more similarities than differences. During normal wound healing, damaged blood vessels release coagulation factors. The resulting fibrin network not only stabilizes the thrombus and results in hemostasis but also serves as provisional ECM, into which fibroblasts and immune cells migrate to rebuild the damaged tissue. A solid tumor mass reaches blood vessels by growth or it attracts blood vessels. Either way, tumor cells infiltrate the EC layer, thereby causing blood components to leak into the surrounding tissue. Among them are coagulation factors that, similar to wound healing, generate a fibrin-rich ECM [412]. Even before blood vessel leakage occurs, tumor cells induce the resident cells to change their behavior. Thus, fibroblasts differentiate into CAFs, which, with their myofibroblastic appearance and increased biosynthesis of ECM matrix components, raise the tension of the tumor matrix and modify the biophysical and biochemical composition of the TME [11]. Stromal cells produce over 90% of a tumor’s ECM mass, mostly core matrisome proteins such as collagens, glycoproteins, and proteoglycans, while the far smaller yet more diverse proportion is from cancer cells [413]. Remarkably, it is precisely these matrix proteins formed by cancer cells that correlate with a poor prognosis for the patient [413]. CAFs are involved in all stages of cancer progression and in the host response to tissue damage by cancer cells [414,415]. They also secrete a large number of cytokines, growth factors, proteoglycans, matricellular proteins, and matrix-degrading enzymes, which promote the growth and mobility of cancer cells. In addition, CAFs control the assembly of a rigid and anisotropic network of collagen I.

Stimulated by cancer cells, resident and invading immune cells also develop into highly active cells that promote tumor progression [53,414,416,417,418,419,420]. Both cancer and immune cells secrete soluble factors such as growth factors (GFs) and cytokines and, thus, induce the differentiation of fibroblasts into CAFs. MMPs affect various functions of the immune system in cancer progression. MMP-9 suppresses the proliferation of T-lymphocytes by shedding the interleukin-2 receptor-α from their surface and, like the MMPs -2 and -14, it impedes the reaction of T-lymphocytes against cancer cells by releasing TGF-β [56]. The sensitivity of NK cells to cancer cells is decreased by the release of the α1 proteinase inhibitor by MMPs -1, -3, -7, -8, and -11 [56]. Moreover, MMPs -7 and -8 affect leucocyte infiltration by cleavage and mobilization of chemokines [56].

The M2-like tumor-associated macrophages (TAMs) support the tumor by producing distinct MMPs [421], such as MMPs -2 and -9, in various tumor entities [422,423,424,425,426,427] with poor prognosis [428]. MMP-14 is upregulated in glioma cell lines by astrocyte-derived IL-6 and increases their migration and invasiveness [429]. By activating MMP-2, MMP-14 can modulate the concentration of alarmin S100A9 and, thus, negatively regulate inflammatory reactions [430,431,432]. In monocytes, the cytoplasmic tail of ICAM-1 interacts with MMP-14, and shedding of the ICAM-1 ectodomain by MMP-14 is essential for efficient endothelial transmigration [433].

Altered ECM synthesis by CAFs and cancer cells results in a fibrotic process termed desmoplasia, in which overshooting collagen production by fibroblast results in a rigid meshwork of type I collagen-rich fibrils. By adhering to them, myofibroblastic CAFs set this meshwork under strong tension and cause an increased interstitial pressure typical of solid tumors [434,435]. Conversely, ECM-cleaving MMPs, typically found during wound healing and in fibrotic processes, determine the turnover of the tumor stroma and reduce contact guidance by fibrillar matrix structures [436]. They are needed by the motile tumor cells to penetrate the dense and tensed collagen network of the TME and tumor collagenous capsule. The triple-helical structure provides a slim stick-like appearance to the collagen molecule and bestows it with an exceptionally high resilience against proteolytic attack, in contrast to the non-triple-helical collagen chains forming the proteolytically highly accessible gelatin after the triple helix is melted. Only a selected set of proteinases, among them MMP-14, is able to cleave triple-helical collagens along a mechanism in which the triple helix is partially melted with the help of an auxiliary domain, such as the hemopexin domain [437,438]. According to their distinct supramolecular arrangement, the different collagen types can be subdivided into, e.g., fibril-forming, network-forming, and fibril-associated collagens. While type I collagen, in association with type III and V collagens form fibrils typically scaffolding the interstitial stroma, type IV collagen shapes a chicken-wire network, which is a structural component of sheet-like BMs [439].

Fibronectin is not only increased quantitatively in the tumor stroma, it is also qualitatively different. The supramolecular fibrin filaments consist of fibrin molecules, which are made of two disulfide-linked chains, which come in different splice variants. Conspicuously, the splice variants with the extradomains (ED) A and B, are predominantly expressed in the tumor stroma. They are otherwise found in fetal tissues during development and wound healing, but are also re-expressed in cancer cells [440]. The fibronectin meshwork interlinks with the collagen network to form a scaffold, into which other ECM proteins are woven. Of special interest are proteins, which are abundant within the intermediate surroundings of cells. Such matricellular ECM proteins (reviewed in [441]) are small leucine-rich proteins (SLRPs), such as decorin and versican [441,442,443] and various other protein families, e.g., CCN proteins, tenascins [444,445,446], SIBLINGs [447], galectins [448], SPARC [449], thrombospondins [450], and periostin [451]. They undergo characteristic changes during tumor progression [452]. Tenascin W appears to be a characteristic marker of the TME [453].

The ECM protein scaffold provides attachment points for cells, which adhere and sense it via cell adhesion molecules, among them integrins. Via integrins, they also exert mechanical forces onto the ECM meshwork, thereby setting it under tension. Stiffness and tension are two biophysical parameters that are sensed by cells and contribute to the characteristic TME. Moreover, mechanical tension releases TGF-β, which is tethered to the ECM in the non-tensed state [454]. TGF-β strongly stimulates differentiation of CAFs, which in turn produce more ECM. Stiffness of the ECM scaffold can be increased by biochemical crosslinking of its molecules to form supramolecular complexes [455]. Thus, in addition to increased deposition of collagen I, the TME and the metastatic niche are enriched in lysyl-oxidases (LOXs) and LOX-like proteins (LOXL), which crosslink collagen molecules via oxidatively derived lysyl side chains [456,457]. Additionally, transglutaminase activity in the TME increases ECM rigidity [457].

ECM rigidity is generally decreased by proteinases such as MMPs. Pericellular MMPs can selectively cleave ECM components in front of an invading cell to open its path through the ECM meshwork. Furthermore, by cleaving the γ-2 chain of laminin-5, MMP-14 and MMP-2 can release an EGF-like fragment that promotes EGFR-dependent cell motility and proliferation [458,459,460]. Accordingly, various cleavage products of ECM proteins are able to serve as soluble cytokines. Hence, the name “matrikine” was coined for them. Growth factors and cytokines, as well as exosomes, are additional means of cell–cell communications within the TME [461].

The acidification of the TME by lactic acid promotes the formation of invadopodia, the trafficking of lysosomes to these structures where they fuse with the plasma membrane, and the production of exosomes [462]. This results in the release of lysosomal proteases and in incorporation of the vacuolar H^+^-ATPase of the lysosomal membrane into the invadopodial membrane, which, together with NHE-1, lowers the pH and favors EMC degradation [462]. NHE1, recruited to invadopodia by phosphorylation of cortactin, acidifies the peri-invadopodial space and, thus, promotes the proteolysis of ECM components by MMPs -2 and -9 [7,463]. NHE1 also stimulates the expression of MMP-14 via ERK1/2 and p38-MAPK signaling in MDA-MB-231 breast cancer cells [464]. By recruiting β1 integrin to the NHE1/NHERF1/p-ezrin signal complex, integrin-linked kinase (ILK) stimulates the proteolytic activity and invasion of invadopodia [465].

The specific topography of collagen fibers at the tumor–stromal boundary is crucial in local invasion [19,466]. Moreover, the stiffness of collagen, increased by LOX-mediated crosslinkages, triggers invasion-promoting signaling in premalignant epithelial cells [467,468]. Mechanical forces that are exerted onto cancer cells by tissue tension or that the cancer cells exert on their surrounding TME are essentially involved in mechanotransduction-induced gene regulation and cancer cell migration and invasion [469,470].

### 5.2. MMPs Generate Bioactive Matrikines during Degradation and Remodeling of the ECM

MMPs can cleave ECM components of the TME to release matrikines. These bioactive peptides regulate tumor progression and metastasis and can be used diagnostically [11,12]. For example, degradation of perlecan by MMPs or cathepsins generates several fragments, in particular, the angiostatic endorepellin [471]. Subsequent proteolytic cleavage of endorepellin by proteases and cathepsin L releases a laminin G-like domain, which binds α2β1 integrin [472].

Additionally, plasma membrane-bound proteins such as the adhesion G protein-coupled receptor B1 (ADGRB1, BAI1), an orphan G protein-coupled receptor protein, can also be cleaved by MMP-14, thereby producing bioactive molecules with matrikine-like function, such as the angiogenesis-inhibiting vasculostatin-40 [473].

Conversely, the activity of MMPs is subject to regulation by matrikines. For example, lamstatin (NC1), the C-terminal domain of the α5 chain of type IV collagen, inhibits tumor cell migration by down-regulating both αvβ3 integrin and MMP-14 [474]. Similarly, hyaluronic acid (HA) oligosaccharide fragments released by glycosidases can modify the expression of MMPs [402,475,476].

## 6. Translational Perspectives: MMPs and Invadopodia Are Worthwhile Targets for Inhibiting Cancer Progression

MMP levels increase with cancer stage and malignancy and are, hence, used as diagnostic and prognostic biomarkers [105,477]. Moreover, the realization that MMPs significantly shape the TME has stimulated the development of tumor-targeting MMP-responsive nanomaterials and nanocarriers for cancer therapy [478]. Such “smart” materials should enable the targeted delivery of drugs, imaging, and theranostic agents on the tissue, cell, and intracellular levels [478]. While this strategy promises effective drug delivery and tumor targeting, its success, either alone or in combination with other therapies, remains to be seen [478].

**Figure 8:**
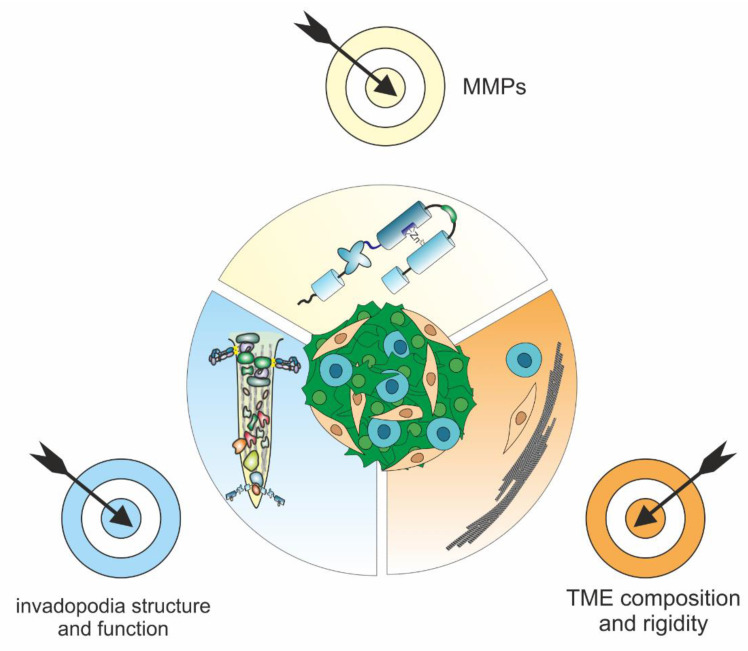
Possible approaches to inhibit metastasis. In addition to MMP inhibitors, therapeutic approaches that aim at the structure and function of invadopodia or at the TME with its increased matrix rigidity are conceivable to inhibit metastasis.

In addition to the use of MMP inhibitors, therapeutic approaches aimed at the structure and function of invadopodia or at the TME with its increased matrix rigidity are also conceivable in order to inhibit metastasis (Figure 8).

Metastasis, the leading cause of death in cancer patients, occurs due to the ability of cancer cells to remodel and break down the surrounding ECM and invade other tissues. Invadopodia correlate with a poor prognosis in various cancers [349]. Consequently, three conceivable routes to curb metastasis may be to target (1) select MMPs, (2) invadopodia, or (2) other components or the biomechanics of the TME. A deeper understanding of the underlying functional mechanisms is beneficial for the development of new markers and therapies.

Although MMPs on the cell surface or in the extracellular compartment are, in principle, druggable, more than 50 different MMP inhibitors have so far proven to be unsuccessful in clinical studies [17,479,480,481]. In a phase I/IIa clinical trial [482] started in January 2018, the drug BT1718 is being studied in adult patients with advanced solid tumors. BT1718 is a ‘bicycle drug conjugate’ that targets and inhibits MMP-14. Upon binding to MMP-14, it is taken up by cancer cells and causes them to die. Other clinical trials on MMPs are described by [483,484]. MMP inhibitors have recently been reviewed by [485].

The biological activity of an MMP depends not only on its catalytic domain but also on so-called exosites on its other domains, e.g., the hemopexin domain that helps to unwind the collagen triple helix. Moreover, the large spectrum of MMP substrates may be another problem, as cleavage of one substrate protein, such as collagen, may help tumor progression, while release of a matrikine from another substrate by cleavage through the same MMP may have an opposite effect on tumor cells [98]. The situation is further complicated by the observation that the same MMP may exert opposite effects on early vs. late stage of tumor progression [98]. Therefore, ideally, inhibitors should be both MMP-specific and substrate-specific and, moreover, time-specific and specific for the subcellular location at which the respective MMP acts pathologically [98]. There is a wide variety of MMP inhibitors of clinical relevance, ranging from hydroxamate-based inhibitors and new-generation hydroxamate-based inhibitors, over non-hydroxamate-based inhibitors to drugs that target alternative binding sites and antibody-based therapeutics, as well as endogenous inhibitors of MMP function [25]. With respect to MMP-14, a monoclonal antibody that blocks MMP-14 abrogates invasion, angiogenesis, and tumor growth in ovarian cancer [40,486].

As compared to MMP inhibition, completely different therapeutic approaches are conceivable that target the structure and function of the invadopodia [487]. After their first observation in Src-transformed fibroblasts, invadopodia have been detected in many invasive human cancer cells such as breast cancer, melanoma, and glioblastoma and correlate with poor prognosis [24,371,488,489,490,491]. However, there is still no invadopodia-targeted cancer treatment. Research is underway on drug repurposing and the inhibition of proteins that are essential to invadopodia, such as signaling pathways, ion channels, or MMPs [24,159,349,392,487,492]. Inhibition of NRTKs in the PTK2B-Src-ABL2-cortactin cascade, in particular of ABL2, effectively inhibits the invadopodia-mediated invasiveness and metastasis of human breast cancer cells in a mouse tumor model [493]. The interaction between PTK2B and FAK in controlling the formation and function of invadopodia suggests the inhibition of both kinases in order to curb metastasis [381].

The Src kinase inhibitor saracatinib prevents invadopodia formation in neck squamous cell carcinoma cells [494] and in a chicken tumor model system [392]. Direct inhibition of invadopodia formation by RNA interference against TKS4 or TKS5 also significantly reduces cancer extravasation in a mouse model [392]. However, knockdown or inhibition of various components of invadopodia of already established secondary micro- or macrometastases does not reduce tumor growth [349]. Even worse, paclitaxel increases both the incidence of invadopodia and the invasiveness of several human cancer cell lines, which raises concerns for its potential clinical use before neoadjuvant therapy or in patients with refractory tumors [495].

The TME with its increased matrix rigidity could be another conceivable target for pharmacologically inhibiting cancer metastasis. Recent progress in targeting the TME in both drug development and clinical trials has been summarized by [496,497]. The role of mechanical forces in cancer has now become clear and sparked the idea of using the physical properties of TME in a targeted manner [498]. Several clinical studies with ECM-normalizing drugs and drugs that target mechanotransduction with a possible effect on MMP-14 function are currently underway [498,499]. Targeting transglutaminase or lysyl oxidase with therapeutics could be options to fight cancer by reversing the biomechanical stiffening of the TME [456,457].

ECM proteins derived from cancer cells or their regulators could be other potential therapeutic candidates [413]. Lumican, a small, leucine-rich proteoglycan, can directly bind to and inhibit MMP-14 and, hence, may limit tumor progression by preventing collagen degradation and invasion [500]. Lumican has an anti-tumor effect in breast cancer, pancreatic cancer, and malignant melanoma by counteracting MMP-14 signaling, among other things [501].

Since the active form of MMP-14 appears to be a better biomarker than its gene expression, several fluorescence- and bioluminescence-based MMP activity reporter constructs have been developed to study the relationship between MMP-14 and the TME in vivo [502,503]. The activities of other MMPs can also be investigated with nanoprobes [504,505]. For example, there is a fluorescent nanoprobe for MMP-2 activity, which is not activated by membrane-tethered MMP-14 [506].

As knowledge of tumor cell dissemination and invasion increases, it becomes evident that a successful strategy to impede metastasis has to envision both adhesion and proteolysis.

## Figures and Tables

**Table 1:** Contribution of MMPs to cancer progression and their main proteolytic activities. Individual MMPs are involved to varying degrees in different stages of cancer progression by activating other MMPs and cleaving ECM components as well as other pericellular molecules. Further MMP substrates that are not directly related to cell–matrix and cell–cell interaction can be found in the text.

MMP	Involved In	Substrates		
		MMPs [47,57]	ECM [15,47,57]	Cell–Matrix and Cell–Cell Receptors [47,57]
MMP-1	EMT [17,35], invasion and metastasis [36]	proMMPs -1, -2, -9	collagens I, II, III, VII, VIII, X, XI, gelatin, elastin, fibronectin, vitronectin, aggrecan, neurocan, brevican, decorin, perlecan, laminin-5, nidogen, CTGF (CCN2), tenascin, SPARC, fibrinogen, fibrin, link protein	
MMP-2	EMT [36]	proMMPs -1, -2, -9, -13, MMP-12	collagens I ^(a)^, III ^(a)^, IV ^(a)^, V ^(a)^, VII ^(a)^, X ^(a)^, XI ^(a)^, gelatin, elastin, fibrillin, fibronectin, vitronectin, aggrecan, laminin, nidogen, tenascin, fibrinogen, fibrin, decorin, link protein	dystroglycan
MMP-3	EMT [34,37], invasion and metastasis [36]	proMMPs -1, -2, -3, -7, -8, -9, -13	non-triple-helical regions of collagens III, IV, V, VII, IX, X, XI, collagen telopeptides, gelatin, elastin, fibrillin, fibronectin, vitronectin, aggrecan, versican, decorin, biglycan, perlecan, laminin, nidogen, fibulin, tenascin, SPARC, osteopontin, fibrinogen, fibrin, link protein, myelin basic protein	E-cadherin [58]
MMP-7	EMT [38], invasion and metastasis [36]	proMMPs -1, -2, -7, -9	collagen IV ^(a)^, non-triple-helical regions of collagens IV, V, IX, X, XI, gelatin, elastin, fibronectin, vitronectin, aggrecan, brevican, versican, decorin, laminin, nidogen, fibulin, tenascin, SPARC, osteopontin, galectin-3, fibrinogen, fibrin, link protein, myelin basic protein	E-cadherin [59], β4 integrin, syndecans -1, -2 [60]
MMP-8	tumor angiogenesis [54]	proMMP-8	collagens I, II, III, gelatin, aggrecan, link protein	
MMP-9	EMT [36], invasion and metastasis [54], tumor angiogenesis [50]	proMMPs -2, -9, -13, ADAMTS-4 ^(b)^	non-triple-helical regions of collagens I, IV, V, XI, XIV, collagens III ^(a)^, IV ^(a)^, V ^(a)^, gelatin, elastin, fibrillin, fibronectin, vitronectin, aggrecan, versican, decorin, biglycan, laminin, nidogen, SPARC, galectins -1 and -3, fibrinogen, fibrin, link protein, myelin basic protein	E-cadherin, β2 integrin, dystroglycan
MMP-10	invasion and metastasis [51], tumor angiogenesis [51]	proMMPs -1, -2, -7, -8, -9, -10, -13	collagens I ^(a)^, III ^(a)^, IV ^(a)^, V ^(a)^, gelatin, elastin, fibronectin, aggrecan, brevican, laminin-5, link protein, fibrinogen	
MMP-11	invasion and metastasis [51]	proMMPs -2, -11	collagen IV ^(a)^, gelatin, fibronectin, aggrecan, laminin	
MMP-12			collagens ^(a)^ I, IV, V, gelatin, elastin fibrillin, fibronectin, vitronectin, aggrecan, decorin, biglycan, laminin, nidogen, SPARC, fibrinogen, fibrin, myelin basic protein	
MMP-13	invasion and metastasis [36]	proMMPs -2, -9, -13	collagens I, II, III, VI, VII, IX, X, XIV, gelatin, fibrillin, fibronectin, aggrecan, brevican core protein precursor, biglycan, perlecan, laminin-γ2, nidogen, CTGF (CCN2), tenascin, large tenascin C, SPARC, fibrinogen	
MMP-14	EMT [39,40], invasion and metastasis [36], tumor angiogenesis [17,55]	proMMPs -2, -8 [61], -13, -14, MMP-14, ADAM9 ^(b)^	collagens I, II, III, gelatin, tropoelastin [62], elastin [62], fibrillin, fibronectin, vitronectin, aggrecan, perlecan, lumican, nidogen, laminins -1, -2, -4, -5, CTGF, CTGF-L (CCN5), Cyr61 (CCN1), tenascin, galectins -1 and -3, fibrinogen, fibrin, myelin basic protein	E-cadherin, N-cadherin, ICAM-1, αV integrin, syndecan-1, syndecan-2 [60], CD44, ICAM-1, DLL1, EMMPRIN
MMP-15		proMMPs -2, -13	collagen ^(a)^ I, NC1 (collagen IV), fibronectin, aggrecan, perlecan, laminin-1, nidogen, tenascin, fibrinogen, fibrin, myelin basic protein	
MMP-16	invasion and metastasis [36]	proMMP-2	collagen III ^(a)^, gelatin, fibronectin, vitronectin, laminin-1, fibrin, myelin basic protein	
MMP-17		proMMP-2, ADAMTS4 ^(b)^	gelatin, fibronectin, laminin-1, chondroitin sulfate proteoglycan, dermatan sulfate proteoglycan, fibrinogen, fibrin, myelin basic protein	N-cadherin
MMP-19		proMMP-19, MMP-9	collagen IV ^(a)^, gelatin, fibronectin, aggrecan, laminin, nidogen-1, tenascin, large tenascin-C, COMP, fibrinogen, fibrin	
MMP-20		proMMP-20 (autolysis)	collagen XVIII ^(a)^, gelatin, aggrecan, laminin, COMP, amelogenin, ameloblastin	
MMP-21			gelatin, aggrecan	
MMP-23			gelatin, fibronectin	
MMP-24		proMMP-2, ADAMTS4 ^(b)^	gelatin, fibronectin, chondroitin sulfate proteoglycan, dermatan sulfate proteoglycan, fibrinogen, fibrin	
MMP-25		proMMPs -2, -9	collagen IV ^(a)^, gelatin, fibronectin, laminin-1, chondroitin sulfate proteoglycan, dermatan sulfate proteoglycan, SPARC, galectin-1, fibrinogen, fibrin, myelin basic protein	
MMP-26	invasion and metastasis [36]	proMMPs -9, -26	collagen IV ^(a)^, gelatin, fibronectin, vitronectin, fibrinogen	
MMP-27		proMMP-27 (autolysis)	gelatin	
MMP-28	EMT [41], invasion and metastasis [36]			NCAM

^(a)^ While MMPs -1, -8, -13, and -14 are true collagenases that can cleave triple-helical collagens, other MMPs can only cleave single collagen chains after unwinding of their triple helix. ^(b)^ Although this is not an MMP, it is listed here because of its related activity.

**Table 2:** Overview of cell–matrix adhesion structures and their involvement in pericellular proteolysis. Cells interact with their surrounding matrix with special adhesome structures. Depending on the function for which different cell types use them, they contain different components. Some adhesome structures possess proteolytic activity. There are also other less common adhesomes, e.g., linear invadosomes and lobopodia, which, for the sake of clarity, are not included in this table [40,228].

Adhesome Structure	Focal Complex [229]	Focal Adhesion [229,230]	Fibrillar Adhesion [231]	Podosome ^(a)^ [229,232,233,234]	Invadopodium ^(a)^ [229,230,232]	Invadosome-like Protrusion [235]	Sealing Zone of Resorption Lacuna [236]
**Occurrence**	adherent cells	adherent cells	adherent cells	rat sarcoma virus (Ras)-transformed fibroblasts, macrophages, immature dendritic cells, osteoclasts, ECs, myoblasts, neural crest cells [233]	invasive cancer cells [237]	lymphocytes [238]	osteoclasts, macrophages, dendritic cells [239]
**Proteolytic activity**	no	no	no	yes: MMP-14 [233]	yes ^(b)^	no? ^(c)^	yes: lysosomal enzymes [240,241]
**Matrix receptors**	β1 and β3 integrins, αV integrins	β1 and β3 integrins, αV integrins	β1 and β3 integrins, αV integrins	β1 and β2 integrins: α2β1, α3β1, α4β1, α5β1, α6β1, αVβ1, αLβ2, αMβ2, αXβ2, αDβ2, αVβ3, β4, β5 [229], CD44 [242]	β1 and β2 integrins: α2, α2β1, α3β1, α4β1, α5β1, α6β1, αVβ1,β2, αLβ2, αMβ2, αXβ2, αDβ2, αVβ3, β4, β5 [229]	integrin αLβ2 [243]	CD44, β3 integrins, αvβ3 [242,244]
**Essential structural components**	phospho-paxillin, FAK, α-actinin, talin [245]	actin, paxillin, FAK, talin, zyxin, vinculin, VASP [245]	dephospho-paxillin, FAK, talin, vinculin, VASP, α-actinin, tensin [245]	actin, vinculin, talin, Arp2/3, myosin IIa, capping protein, TKS4/5, Cdc42, Src [234]	actin, Arp2/3, cortactin, N-WASp, Nck1, cofilin, TKS5 [246]	actin, talin, vinculin [243]	actin, vinculin, talin, paxillin, zyxin, Arp2/3, N-WASp, myosin X, Arp2/3, capping protein, TKS4/5 [236]
**Diameter [µm]**	0.5–1 [247]	1–5 [247]	>5 [247] ^(d)^	0.2–2 [248,249,250]	0.5–2 [251],8 [232]	0.2–1 [238]	>14 [239], 95–130 [242] ^(e)^
**Protrusion depth [µm]**	-	-	-	0.2–0.5 [248,249,250]	>10 [243],>60 [232]	<10 [243]	- ^(f)^
**Lifetime [min]**	2–3 [247]	20–90 [252]	very stable [247]	2–12 [232,253]	>10 [243],>60 [232]	<10 [243]	8–360 [254]
**Number per cell**		<400 [246], variable ^(g)^ [255]		20–100 [232]	1–10 [232]	10–100 ^(h)^ [243]	variable ^(i)^ [255]
**Function**	Cell–matrix contact	cell–matrix contact	Cell–matrix contact	Cell–matrix contact, ECM degradation, sensing of substrate rigidity and topography, antigen sampling, myoblast fusion [233]	cell–matrix contact, ECM degradation, sensing of substrate rigidity and topography	biomechanical scanning, cell–cell interaction, diapedesis [235]	cell–matrix adhesion, sealing of the bone resorption lacuna [239]

^(a^^)^ Depending on the cell type, podosomes and invadopodia can form higher-order invadosome structures, such as linear, array, single, rosette, belt, and ring assemblies [256,257]. ^(^^b)^ Invadopodia in cancer cells resemble podosomes in normal cells but are more degradative [258]. ^(c^^)^ So far, no proteases have been observed in ILPs. Although they are not necessary for transcellular pore formation, if present, they could influence adhesion and signal dynamics and be involved in breaching the BM [243]. ^(d^^)^ Elongated with a typical axial ratio >7 [259]. ^(e^^)^ Physiologically, sealing zones on the bone are 10 or more micrometers in size, while, in culture, they can reach hundreds of micrometers [239]. ^(f^^)^ Sitting flat on the bone substrate, the sealing zone structure inside the cell is approx. 4 µm thick [260]. ^(g^^)^ The number depends on the matrix stiffness [255]. ^(h^^)^ Endothelial invaginations by leukocyte protrusions [238] ^(^^i)^ The number depends on bone roughness [239].

## Abbreviations

ABL2	Abelson murine leukemia viral oncogene homolog (Arg, Abl-related gene)
ADAM	a disintegrin and metalloproteinase
ADAMTS	a disintegrin and metalloproteinase with thrombospondin motifs
ADGRB1	adhesion G protein-coupled receptor B1
Arp2/3	actin related protein 2/3 (complex)
AP-2µ2	adaptor protein-2 subunit µ2
Ask1	apoptosis signal-regulating kinase 1
BAI1	brain-specific angiogenesis inhibitor 1
BLACAT1	bladder cancer-associated transcript-1
BM	basement membrane
CAF	cancer-associated fibroblast
CAP-G	capping actin protein, gelsolin-like
CCN	Cyr61-CTGF-NOV family of matricellular proteins
CDC42	cell division control protein 42 homolog
CDCP1	CUB-domain-containing protein 1
Cdk5	cyclin-dependent kinase 5
CLCN7	chloride voltage-gated channel 7
CLIC4	intracellular chloride channel 4
COMP	cartilage oligomeric matrix protein
CTGF	connective tissue growth factor, CCN2
CTGF-L	connective tissue growth factor ligand, CCN5
Cyr61	Cysteine-rich angiogenic inducer 61, CCN1
DDR1	discoidin domain receptor 1
DKK-3	Dickkopf-related protein-3
DLL1	Delta-like 1
EC	endothelial cell
ECM	extracellular matrix
EGF(R)	epidermal growth factor (receptor)
EGR-1	early growth response protein-1
EMT	epithelial-to-mesenchymal transition
ER	endoplasmic reticulum
ER-β	estrogen receptor β
ESCRT	endosomal sorting complex
EVL	Ena/vasodilator-stimulated phosphoprotein (VASP)-like
FAK	focal adhesion kinase
FYCO1	FYVE and coiled-coil domain-containing protein 1
FIH-1	factor inhibiting HIF-1
GRASP55	Golgi reassembly stacking protein 55
HA	hyaluronic acid
HB-EGF	heparin-binding epidermal growth factor
HIC-5	hydrogen peroxide-inducible clone 5 protein (TGFB1I1)
HIF	hypoxia induced factor
ICAM-1	intercellular adhesion molecule-1
IκBα	nuclear factor of kappa light polypeptide gene enhancer in B-cells inhibitor, alpha
IL	interleukin
ILK	integrin-linked kinase
ILP	invadopodia-like protrusion
IQGAP1	Ras GTPase-activating-like protein
KIF	kinesin superfamily protein
LAMTOR1	late endosomal/lysosomal adaptor, MAPK, and mTOR activator 1
LOX(L)	lysyl oxidase (-like)
mDia2	Diaphanous-related formin protein 2
MAPK	mitogen-activated protein kinase
Mena/VASP	protein-enabled homolog/vasodilator-stimulated phosphoprotein
Mi-2/NuRD	nucleosome remodeling deacetylase
Mint3	Munc18-1-interacting protein 3
MMP	matrix-metalloproteinase
mTOR	mechanistic target of rapamycin
NC1	non-collagenous domain-1
NCAM	neural cell adhesion molecule
NCK1	non-catalytic region of tyrosine kinase adaptor protein 1
NET	neutrophil extracellular trap
NHE1	Na^+^/H^+^ exchanger 1
NHERF1	Na^+^/H^+^ exchanger 1 regulating factor 1
NOV	nephroblastoma overexpressed, CCN3
NRTK	non-receptor tyrosine kinase
N-WASp	neural Wiskott–Aldrich syndrome protein
PAK	p21-activated kinase
PARK7	Parkinsonism-associated deglycase
PCSK6	proprotein convertase subtilisin/kexin type 6
PARP	poly (ADP-ribose) polymerase
PDZ	post synaptic density protein, Drosophila disc large tumor suppressor (Dlg1), and zonula occludens-1 protein domain
PI3K	phosphatidylinositol 3-kinase
PI(3,4)P2	phosphatidylinositol-3,4-bisphosphate
PKC	protein kinase C
PTK2B	protein tyrosine kinase-2β, Pyk2
PX	phox homology
RAB7	Ras-related protein 7
Ras	rat sarcoma virus
RECK	reversion-inducing cysteine-rich protein with Kazal motifs
RhoA	Ras homolog family member A
SDF	stromal cell-derived factor
SERPINE2	serine proteinase inhibitor, clade E, member 2
Src	sarcoma proto-oncogene tyrosine-protein kinase
SNAI1	snail family transcriptional repressor-1
SPARC	secreted protein acidic and rich in cysteine, osteonectin
TAZ	transcriptional coactivator with PDZ-binding motif
TGF-β	transforming growth factor β
TGFB1I1	transforming growth factor β-1-induced transcript 1 protein (HIC-5)
TIMP	tissue inhibitor of metalloproteinase
TKS4/5	tyrosine kinase substrate (scaffold) protein with four/five SH3 domains
TME	tumor microenvironment
TNF-α	tumor necrosis factor-α
UBTD1	ubiquitin domain-containing protein 1
uPA(-R)	urokinase plasminogen activator (surface receptor)
VASP	vasodilator-stimulated phosphoprotein
WASp	Wiskott–Aldrich syndrome protein
WAVE	WASp and verprolin homolog
Wnt	Wingless-related integration site
YAP	Yes-associated protein 1
ZEB	zinc finger E-box binding homeobox
